# Multi-plateau high-harmonic generation in liquids driven by off-site recombination

**DOI:** 10.1038/s41566-025-01805-y

**Published:** 2025-11-19

**Authors:** Angana Mondal, Ofer Neufeld, Tadas Balčiūnas, Benedikt Waser, Serge Müller, Mariana Rossi, Zhong Yin, Angel Rubio, Nicolas Tancogne-Dejean, Hans Jakob Wörner

**Affiliations:** 1 Laboratory of Physical Chemistry, ETH Zürich, Zürich, Switzerland; 2 Faculty of Chemistry, Technion Israel Institute of Technology, Haifa, Israel; 3 Max Planck Institute for the Structure and Dynamics of Matter, Hamburg, Germany; 4 International Center for Synchrotron Radiation Innovation Smart, Tohoku University, Sendai, Japan; 5 Center for Computational Quantum Physics (CCQ), The Flatiron Institute, New York, NY USA

**Keywords:** Supercontinuum generation, Ultrafast photonics

## Abstract

Non-perturbative high-harmonic generation has recently been observed in the liquid phase, and the underlying mechanism was shown to be different from that in gases and solids. Liquid-phase high-harmonic generation is currently understood in terms of a recollision mechanism with electron trajectories limited by electron scattering. The cut-off energy and its independence of the driving laser parameters are reproduced by this mechanism. However, when the driving laser intensity is increased, no extension of the cut-off energy is observed, which contrasts with the general expectations from most nonlinear media. Here we observe the appearance of a second plateau in high-harmonic generation from multiple liquids (water, heavy water, propanol and ethanol) and explore its origin. From the combined analysis of experimental, computational and theoretical results, we find that electrons recombining at neighbouring molecular sites instead of the ionization site are responsible and verify this feature through the characteristic dependence of the second-plateau yield on the ellipticity of the driving field. We find that the second plateau is dominated by electrons recombining at the first or second solvation shell, relying on hole delocalization. Theoretical results predict the appearance of yet higher plateaus, indicating a general trend. Our work establishes a previously unexplored physical phenomenon in the highly nonlinear optical response of liquid.

## Abstract (web summary)

The second plateau in high-harmonic generation from liquids is due to off-site recombination of electrons, facilitated by the spatial delocalization of electron–hole wavefunctions.

## Main

High-harmonic generation (HHG) is a non-perturbative nonlinear optical process that occurs when matter is irradiated by intense laser pulses. The phenomenon was initially discovered in noble gases in the 1980s^1,2^, followed by its solid-state version in 2011^3^, liquid media in 2018^4^, and has since been widely studied and explored in a plethora of systems and conditions^5–14^. In the gas phase, HHG is well understood as arising from a process in which electrons are (i) tunnel-ionized from their parent molecule, (ii) accelerated in the continuum by the intense laser and (iii) coherently recombine with the parent molecule to emit high-energy radiation^15,16^. A hallmark of this simple physical picture is that it correctly predicts the main spectral and temporal features of the emission, especially its cut-off—the energy above which the yield begins to exponentially decay—which scales parabolically with the driving laser field amplitude and wavelength. This physical interpretation has paved the way to multiple applications ranging from ultrafast spectroscopy^17–21^, coherent extreme ultraviolet pulse shaping^22,23^ and attosecond pulse generation^24–26^.

More recently, HHG has been established and extensively explored in the solid state^6,27–31^, where an equivalent multi-step picture can be formulated in *k*-space after applying Bloch’s theorem^32–35^. The *k*-space trajectory picture predicts a cut-off energy that scales linearly with both the driving wavelength and field amplitude^27^. In principle, also real-space trajectories can be formulated in solids^36–40^, which include also rescattering channels and recombination on various lattice sites^41–43^. However, owing to the wide delocalization of the Bloch states in real space, that is not always a helpful picture^44,45^, for example, requiring recombination of electrons and holes many unit cells apart^34^. Solids were also shown to produce multiple plateau structures arising from their multi-band nature^7,33,46–51^ (just as multiple plateaus can appear in gas-phase HHG if several molecular orbitals are involved)^52,53^. Quite generally, HHG in both solids and gases follows a universal physical principle: when progressively increasing the intensity while driving a nonlinear medium, a higher-order nonlinear response should appear as long as the system has not entered a saturation regime^54,55^ (that is, if the ionization rate has not surpassed a certain threshold per molecule, typically on the order of a single electron, or reached the damage threshold where chemical bonds start breaking up^56^). This principle should be respected regardless of the specific details of the medium, or even the nature of the interaction and its physical mechanism. Indeed, even in some unique cases such as Cooper minima^57^ that cause a locally suppressed response, stronger laser driving eventually leads to appearance of higher harmonic orders.

**Fig. 1:**
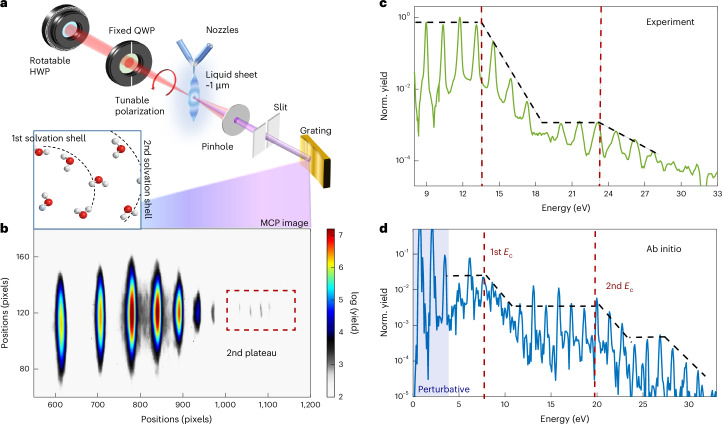
Experimental set-up for liquid-phase HHG and observation of second plateaus in experiment and theory. **a**, Schematic of the experimental set-up. Tunable-wavelength (here 1,800 nm) laser pulses are focused on a flat liquid jet to generate high harmonics. The harmonics pass through a slit, are dispersed by a grating and detected with an MCP-phosphor-screen-camera system. Inset: a schematic of the first and second solvation shells marked by the dashed black arcs. **b**, Raw MCP image of high-harmonic spectrum observed from D_2_O liquid acquired at 4.8 × 10^13^ W cm^−^^2^, showing the presence of a second-plateau feature. **c**, Experimentally observed high-harmonic spectrum from D_2_O liquid acquired at 4.8 × 10^13^ W cm^−^^2^. In addition to a first plateau, a second plateau is observed from 19 eV upwards, with a cut-off energy (*E*_c_) at ~23 eV. **d**, Harmonic spectrum of liquid H_2_O from ab initio simulations^59^ at 1,800 nm and 5 × 10^13^ W cm^−^^2^ (intensity averaged), showing a second plateau with cut-off *E*_c_ at ~20 eV. Red-dashed lines mark cut-off energies, defined at the onset of exponential decay within one-harmonic uncertainty. Norm., normalized.

By contrast, non-perturbative HHG in the liquid phase has only recently been measured^4^ and explained as arising from a trajectory-based picture limited by electron–molecule scattering^8^. This causes the cut-off energy to effectively be independent of the driving wavelength and intensity^8,58,59^, which instead mainly depends on the electron mean-free path (MFP)^8^. Similar physics is expected in amorphous solids^49^ (with the main difference being that liquids also exhibit strong structural dynamics). This result is in contradiction with the general physical expectation, as the driving parameters are far from the saturation limit, and re-absorption does not change the main HHG spectral features. Thus, it remains unclear where the missing high-order response is, or why it is missing, as the laser intensity and wavelengths are increased. Here we experimentally measure HHG in a variety of liquids using methodologies that provide an enhanced signal-to-noise ratio and dynamical range compared with previous measurements^4,8,60^. We report the emergence of a second plateau in liquid HHG, which accounts for the previously unobserved higher-order response. To support experimental findings, we perform state-of-the-art ab initio calculations and establish that it is a robust and generic feature of liquid-phase HHG. We formulate an extended electron-trajectory picture for the laser-driven ultrafast dynamics that qualitatively captures the main features of the second plateau—its spectral range, cut-off scaling and temporal characteristics—and show that it originates from electrons that recombine with neighbouring molecules. Nearest-neighbour (NN) recombination trajectories are shown to be the dominant contributor to the emission window in between the two plateaus and within the second plateau, whereas next-NN (NNN) (that is, electrons recombining at the second solvation shell shown in Fig. 1a, inset) also contribute to the second plateau emission. This mechanism leads to a second-plateau cut-off that scales weakly with the laser wavelength and intensity (similar to the first plateau), but also exhibits a characteristic anomalous dependence of the harmonic yields on the driving laser ellipticity, which we confirm with experiments and theory. Ab initio simulations predict yet higher-order plateaus that we suspect are connected to higher-order recombination-scattering processes involving more distant molecular sites (which currently are not resolvable in the experiment). These HHG mechanisms are different in nature compared with those in both gas and solid phases, thus paving the route to ultrafast spectroscopy of electron dynamics in solution. We anticipate that these mechanisms can be exploited to retrieve effective intermolecular separations, and probe the delocalization of electronic wavefunctions in amorphous media^61^, the hybridization of solute and solvent electronic wavefunctions, as well as their attosecond dynamics.

## Results

### Experimental results

HHG was measured from a liquid flat jet of ~1 μm thickness generated by 2 colliding cylindrical jets of ~50 μm diameter^62^ (Fig. 1a). The thin flat jets not only allow us to directly measure the bulk-liquid response and to separate contributions to HHG coming from evaporated molecules near the liquid surface (see Methods for further details), but also to acquire data with high signal-to-noise ratio. HHG was driven in the liquid flat jet with a 1 kHz Ti:sapphire laser coupled with an optical parametric amplifier (HE-Topas), varying the laser amplitude, wavelength and polarization state. Harmonics were measured with an extreme ultraviolet spectrometer consisting of a grating and micro-channel plate (MCP)-based detector. Details of the experimental apparatus can be found in Methods.

**Extended Data Fig. 1:**
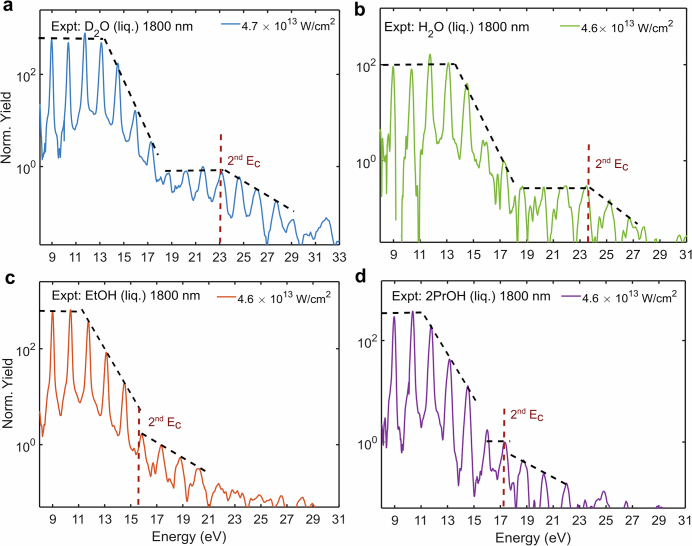
Observation of second plateau at 1,800 nm. Experimental HHG spectra from liquid D_2_O (**a**), H_2_O (**b**), ethanol (**c**) and isopropanol (**d**) at 1,800 nm driving wavelength, all showing second plateaus, indicating the generality of the phenomenon.

**Extended Data Fig. 2:**
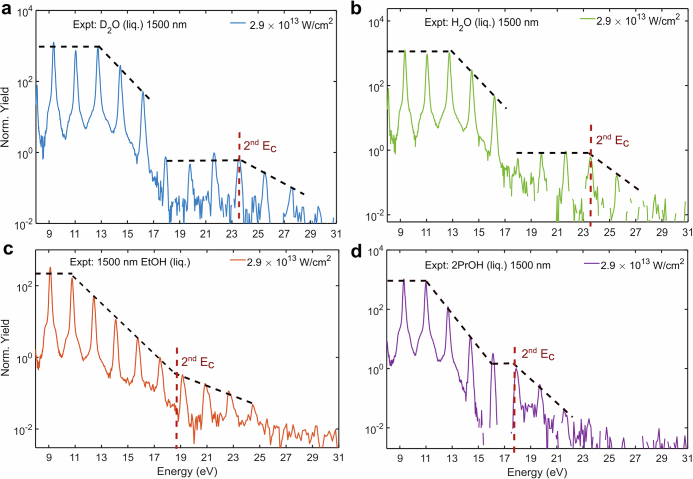
Observation of second plateau at 1,500 nm. Experimental HHG spectra from liquid D_2_O (**a**), H_2_O (**b**), ethanol (**c**) and isopropanol (**d**) at 1,500 nm driving wavelength, all showing second plateaus, indicating the generality of the phenomenon.

Figure 1b,c presents our main experimental observation—in HHG from liquid D_2_O under ambient conditions^63,64^, we observe two distinct HHG plateaus. Similar results have been obtained for H_2_O and alcohols (Extended Data Fig. 1), also at other wavelengths (Extended Data Fig. 2). The second plateau appears at 2–3 orders of magnitude diminished yield compared with the first plateau, and about 5 eV beyond the cut-off energy of the first plateau. In between the plateaus, we observe an ~5-eV-wide exponential-decay region which we refer to as the inter-plateau transition. We note that the cut-off energy of the first plateau is fully consistent with previous measurements and theory^4,8,59,65^, and is roughly independent of the driving wavelength and intensity. Thus, in the absence of appropriate dynamic range, upon driving the medium harder, one would get the appearance of a saturated cut-off energy. Only when discerning signals that are about ~500 times weaker is the missing higher-order response discerned.

**Fig. 2:**
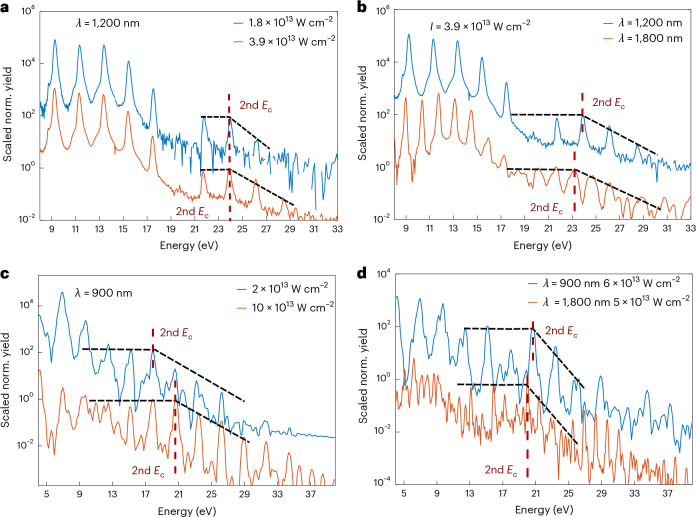
Intensity and wavelength dependence of the second plateau in HHG from liquids. **a**, Intensity dependence: experimental HHG spectra measured in D_2_O liquid at varying driving intensity at 1,200 nm. **b**, Wavelength dependence: experimental liquid D_2_O HHG spectra measured at 1,800 nm and 1,200 nm at similar driving peak intensity. **c**, Intensity dependence: ab initio simulations of intensity-dependent HHG spectra driven at 900 nm in liquid H_2_O. **d**, Wavelength dependence: ab initio HHG simulations for 900 nm and 1,800 nm wavelength for liquid H_2_O at the same driving intensity. This data establishes a very weak wavelength and intensity dependence of the second-plateau cut-off energy both in experiments and theory. All spectra have been normalized with respect to the maximum within the 18–35 eV range. Normalized spectra recorded with lower driving intensities or lower wavelengths, respectively, have been shifted upwards for visualization purposes. For additional data, see Supplementary Fig. 8.

Figure 2a,b presents further experimental investigations, showing that the cut-off of the second plateau is weakly dependent on driving wavelength and intensity, similar to the first plateau (suggesting a qualitatively similar mechanism). We also note that an identical nonlinear response exists in liquid water (H_2_O); however, we have found that D_2_O provides cleaner spectra (most likely owing to its higher viscosity^66^ and isotopic effects^67^). For further details on the determination of the second cut-off energy, see Supplementary Section 7.

### Theoretical simulations and mechanistic interpretation

**Fig. 3:**
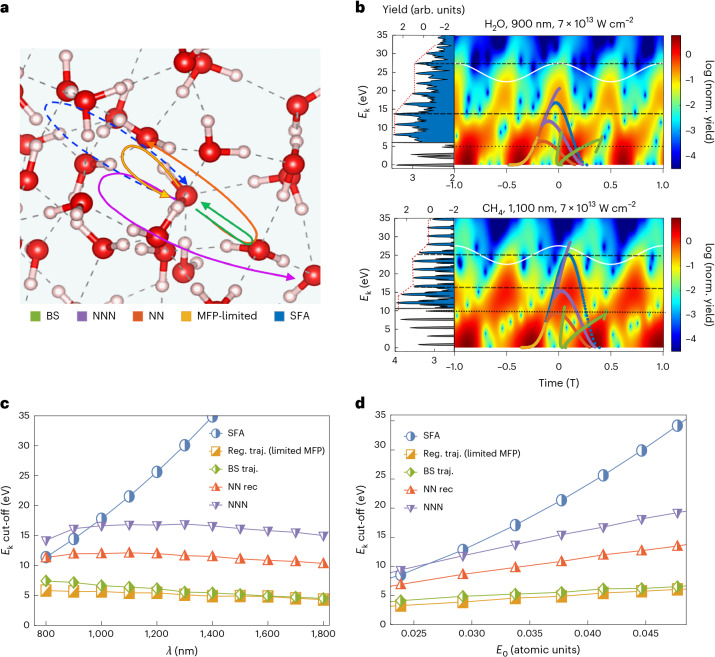
Electron trajectories contributing to HHG from liquids. **a**, Short trajectories within the MFP that recombine on-site (yellow) form the first plateau. Longer trajectories may recombine on neighbouring sites—with lower probability—located in the first solvation shell (orange, contributing to the first plateau/inter-plateau region) or second shell (purple, dominant source of the second plateau). Backscattering (green) is negligible, and long on-site SFA-like trajectories are inconsistent with the second plateau. **b**, Time–frequency analysis showing the temporal dependence of emitted photon energies for liquid H_2_O (top, polar liquid), liquid methane (bottom, apolar liquid), for exemplary laser conditions, obtained from ab initio calculations. Insets on the left of **b** show the different plateau structures in the harmonic spectra, normalized to electronic kinetic energies upon recombination. Each plot is overlaid with the electron kinetic energies and emission times from the different possible trajectories: MFP-limited trajectories contributing to the first plateau (yellow), NN recombination (orange), NNN recombination at the second solvation shell (purple), BS (green) and unrestricted gas-phase SFA trajectories (blue), calculated with the semi-classical model under the same conditions as the ab initio simulations. **c**, Semi-classical scattering model wavelength dependence of the second-plateau cut-off energy. **d**, Semi-classical scattering model intensity independence of the second cut-off energy. Reg. traj., regular trajectory; rec, recombination.

We calculated the liquid HHG spectra relying on time-dependent density functional theory (TDDFT)^68^ using finite clusters^59^ within the Octopus code^69^. This approach assumes that the nuclei remain frozen during their interaction with the laser pulse (which should be valid on few-femtosecond timescales). This renders ab initio simulations for H_2_O and D_2_O essentially equivalent at the microscopic level. Further details on the theoretical approaches are given in Methods and Supplementary Section 1. Figure 1d presents exemplary numerical results in similar conditions to the experiment in Fig. 1c, showing a second HHG plateau appearing with similar attributes and energy scales. In particular, the average yield separation between plateaus and the inter-plateau width are consistent between theory and experiment. This allows us to rule out macroscopic effects as a possible origin for the second plateau (as shown for the first plateau^8^), since the theory is purely microscopic in nature. Further, ab initio calculations agree with the measured weak scaling of the second-plateau cut-off with driving wavelength and intensity (Fig. 2c,d). These conclusions hold in several liquid media and conditions, which have been tested both experimentally in ethanol and isopropanol (Extended Data Figs. 1 and 2), as well as numerically in liquid methane (Fig. 3b) and ammonia (Supplementary Section 4).

Remarkably, theoretical calculations also predict the appearance of an even higher-order third plateau (Fig. 1d), which arises at yet higher photon energies, suggesting a certain universality in the physical mechanism whereby the order of the nonlinear response appears to saturate, but in fact higher harmonics are emitted in exponentially suppressed higher plateaus. However, while ab initio simulations predict this feature, in practice, its observation may be hindered by strong dephasing and scattering effects in liquids, which are not fully accounted for in the simulations and are expected to further suppress coherent emission from long trajectories. The third plateau yield likely falls below the current dynamic range of our experiment.

Having established the existence and microscopic origin of a second plateau in liquid HHG for different liquids and laser conditions, we now explore its physical origin. Given the success of electron-trajectory-based models in HHG in general, and in describing the first plateau in liquid HHG, the second plateau might also be characterized by a set of unique electronic trajectories (which also maintain optical coherence as required from HHG). Owing to the lack of translational symmetry and applicability of the Bloch theorem in liquids (which prevents a consistent definition of a reciprocal lattice), a real-space picture that can still take into account the local structural arrangement and short-range symmetry of liquids is preferrable to a *k*-space one. The main question is then which trajectories could be responsible for emerging higher plateaus. We therefore analyse possible candidates using extended semi-classical models.

**Fig. 4:**
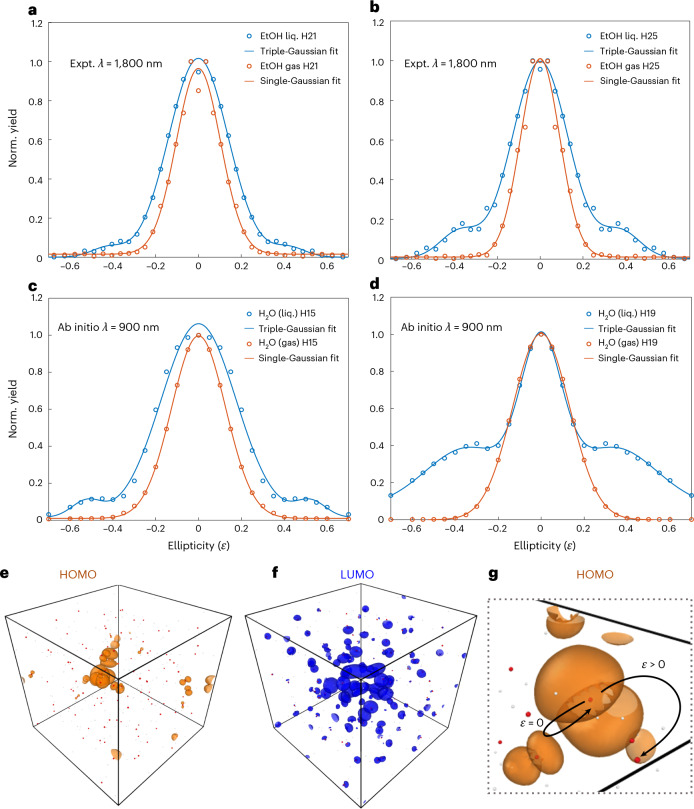
Ellipticity dependence of harmonics in the second plateau of HHG from liquids. **a**,**b**, Experimental comparison of harmonic yield as a function of ellipticity for harmonic orders 21 (**a**) and 25 (**b**) (~14.5 eV and ~17.2 eV, respectively) in gas and liquid phase ethanol at 1,800 nm and 2.4 × 10^13^ W cm^−^^2^. The gas-phase data has been measured by shifting the liquid jet by 300 μm from the point of collision of the 2 cylindrical jets. **c**,**d**, Comparison of harmonic yield as a function of ellipticity for harmonic orders 15 (**c**) and 19 (**d**) (~20.7 eV and ~26.2 eV, respectively) in liquid and gas phase H_2_O, calculated through ab initio simulation at 900 nm and 4 × 10^13^ W cm^−^^2^. For the gas phase, the expected single Gaussian captures the dependence of the harmonic yield as a function of the laser ellipticity (owing to on-site recombination). For liquids, we observe multiple Gaussian features indicating the off-site recombination for second-plateau harmonics. **e**,**f**, HOMO (**e**) and LUMO (**f**) states obtained from snapshots of molecular dynamics simulations of liquid water, indicating a broad delocalization of the LUMO states, which allows for off-site recombination (Supplementary Section 1). **g**, Zoomed-in view of a snapshot of the delocalized HOMO, identifying off-site recombination for elliptically polarized (*ϵ* > 0) light. LUMO, lowest-unoccupied molecular orbital; Expt., experimental; liq., liquid.

**Extended Data Fig. 3:**
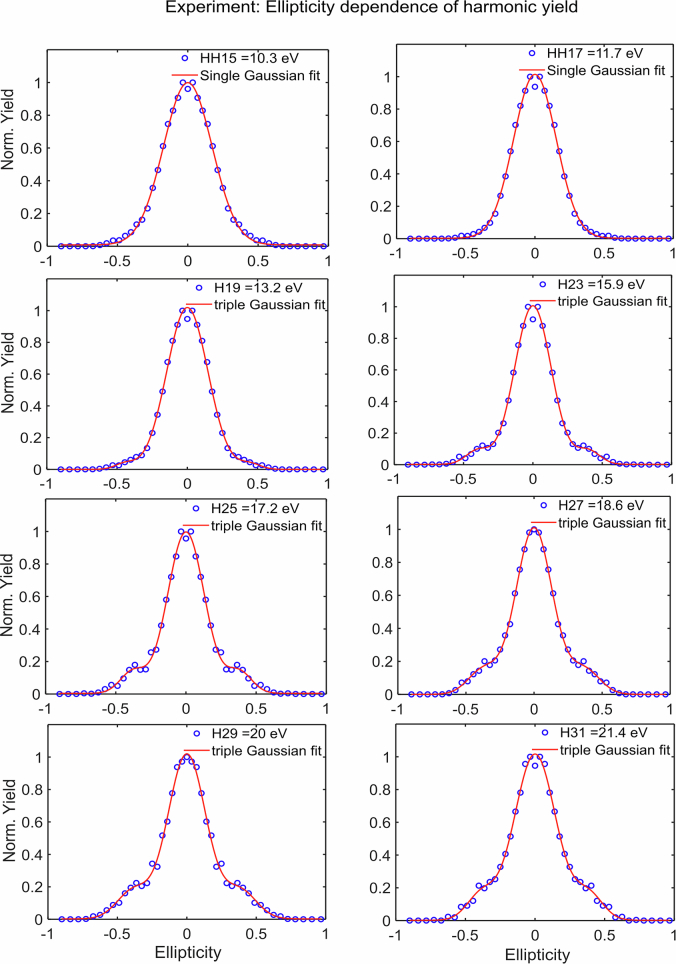
Experimental ellipticity dependence of high-harmonics in liquid ethanol. Experimentally observed ellipticity dependence of high-harmonic yields for liquid ethanol using 1,800 nm wavelength at 2.4 × 10^13^ W cm^−2^.

**Extended Data Fig. 4:**
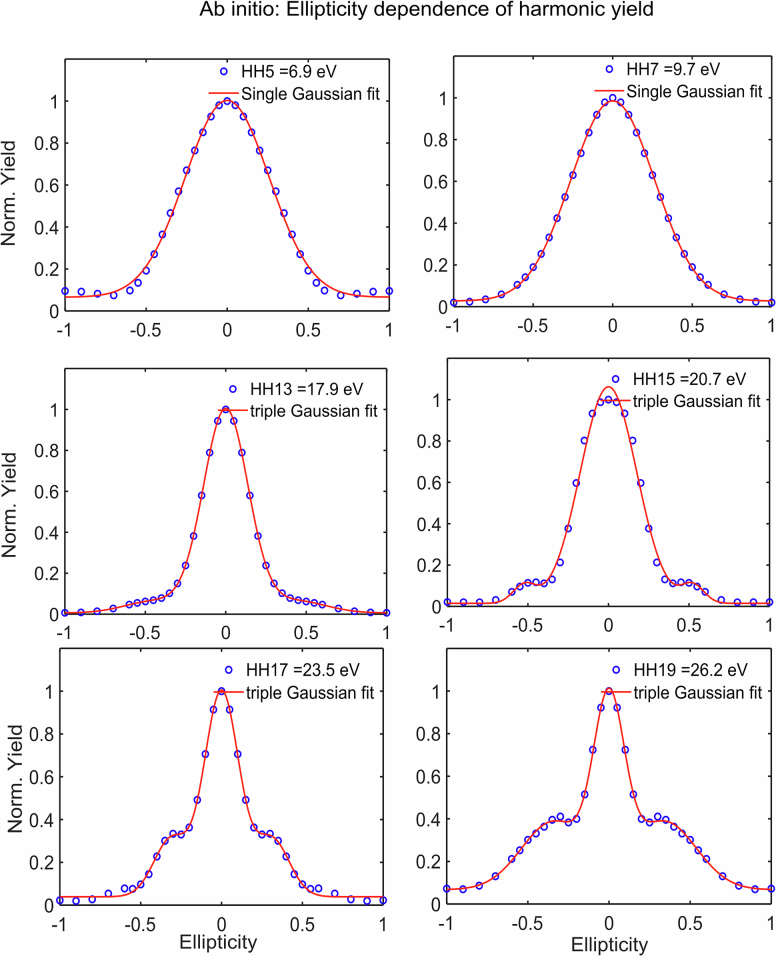
Ellipticity dependence of high-harmonics in liquid water from ab initio simulations. Ellipticity dependence of high-harmonic yields obtained from ab initio simulations for liquid water at 900 nm at 4 × 10^13^ W cm^−2^.

**Extended Data Fig. 5:**
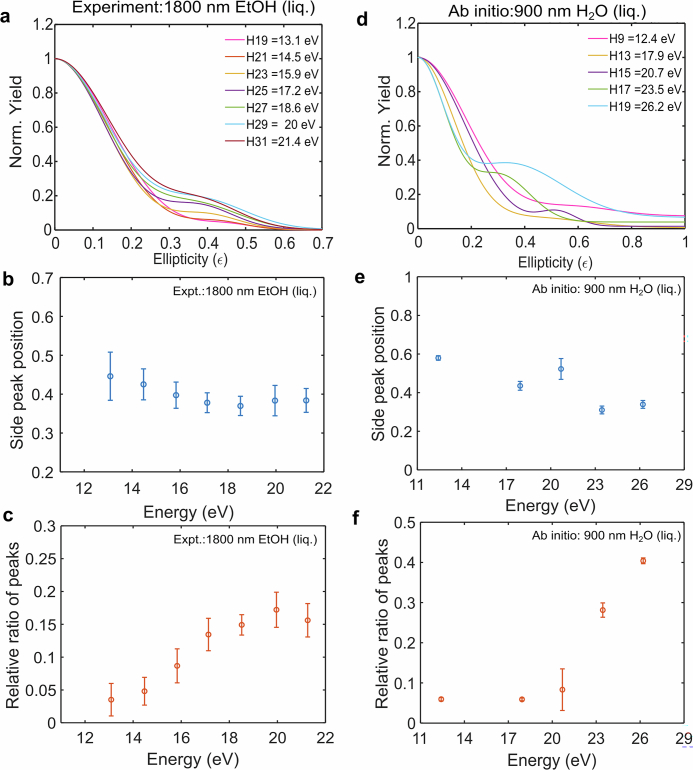
Detailed analysis of the ellipticity dependence of second-plateau harmonics in liquids. Detailed analysis of the ellipticity dependence of measured (left) and calculated (right) second-plateau harmonics. (**a**) Curves fitted to the experimental ellipticity dependence of high-harmonic yields beyond 1st cut-off energy (~ 11.5 eV) for liquid EtOH driven at 1,800 nm at 2.4 × 10^13^ W cm^−2^. (**b**) Extracted side-peak position and (**c**) ratio of the side-peak amplitude divided by that of the central peak at ellipticity = 0 (linear polarization). (**d**) Curves fitted to the calculated ellipticity dependence of high-harmonic yields emitted from H_2_O clusters driven at 900 nm at 4 × 10^13^Wcm^−2^. (**d**) Extracted side-peak position and (**e**) ratio of the side-peak amplitude divided by that of the central peak at ellipticity = 0 (linear polarization). The triple Gaussian fit did not converge for the calculated harmonic of photon energy 15.2 eV, therefore the data point at 15.2 eV has been excluded from Extended Data Fig. 4. Error bars in panels (**b**), (**c**), (**e**), and (**f**) indicate the uncertainties of the fitted parameters within a 95% confidence interval.

The first candidate are the ‘standard’ gas-phase-like electron trajectories, which were assumed to be suppressed by scattering such that they do not contribute to the first plateau^8,65^. We describe these trajectories analytically within the three-step model^15^, while adapting the different steps to the liquid phase, which includes scattering effects described through MFP limitations of the electronic motion. At the moment of ionization, *t* = *t*_i_, an electron is excited from its parent molecule to the liquid-dressed continuum. The electron then propagates classically under the external laser field and can then recombine at *t* = *t*_f_. We neglect the ionic potentials within the strong-field approximation (SFA), which allows for an analytical formulation:

1 $$x(t)=\frac{q{E}_{0}}{{m}_{\rm{e}}{\omega }^{2}}[\cos (\omega t)-\cos (\omega {t}_{{\rm{ion}}})+\omega (t-{t}_{{\rm{ion}}})\sin (\omega {t}_{{\rm{ion}}})],$$

where *E*_0_ is the laser field’s peak amplitude, *q* and *m*_e_ are the electron charge and mass, and *ω* is the laser carrier frequency. By solving equation (1) and demanding that *x*(*t*_f_) = 0 (for *t*_f_ > *t*_i_), we obtain the sets of short and long trajectories responsible for gas-phase HHG. Further restricting the maximal electron excursion distance to be smaller than the typical MFP at those electron energies (*l*_max_), we obtain the measured first-plateau cut-off independence on the laser wavelength and very weak dependence on laser intensity^8^. Next, in considering the second plateau, we can imagine that the set of longer, higher-energy trajectories that were assumed to scatter owing to their length exceeding *l*_max_ might still contribute to HHG, but with a much reduced cross section, leading to a second plateau. However, these trajectories are incompatible with our measurements and calculations for several reasons: (i) The obtained energy ranges for the second plateau do not match those predicted by this set of long trajectories, often differing by ~10 eV. (ii) The cut-off predicted by these trajectories is strongly dependent on the driving wavelength and electric-field amplitude (quadratic in both), inconsistent with the results shown in Fig. 2. (iii) The time–frequency characteristics of these trajectories do not match those coming from ab initio calculations (Fig. 3b,d,e and its deviation from the typical bell-shaped curves obtained from SFA gas-like trajectories). (iv) These trajectories should show a single-Gaussian dependence on the driving laser ellipticity with a width similar or close to that of the gas-phase decay, because the mechanism would be identical^4,70^. We therefore studied the ellipticity dependence of the second-plateau harmonics, both experimentally and through ab initio calculations, and compared the liquid- to the gas-phase results in Fig. 4 (with complete datasets shown in Extended Data Figs. 3–5). Whereas the gas-phase results show the expected Gaussian shape, the liquid-phase results differ markedly. Their ellipticity dependence is best captured with a multi-Gaussian fit and an increased width of the central Gaussian. Moreover, while the position of the side peak in the ellipticity dependence does not strongly depend on the emitted photon energy, the amplitude of the side peak markedly increases with energy, both in experiments and calculations (Extended Data Fig. 5). A full comparison of the first- and second-plateau harmonics is presented in Extended Data Figs. 3 and 4 and Supplementary Figs. 9–11. The observed multi-Gaussian dependence in the liquid phase is indicative of additional underlying recombination pathways beyond the central Gaussian distribution. Note that an anomalous ellipticity dependence can also be observed in gas-phase systems^71^, but with a different characteristic to the side peaks observed here, and only in a particular energy range close to a resonance (while in our case the effect is robust to intensity and wavelength changes, indicating a non-resonant phenomenon). Overall, these results allow us to rule out ‘standard’ gas-phase-like trajectories as a source of second-plateau emission. More importantly, the ellipticity data and the ab initio time–frequency analysis clearly establish that the second plateau originates from a physical mechanism that is different from the one creating the first plateau.

We further consider a set of trajectories whereby the liberated electron is driven in the liquid-dressed continuum, but instead of returning to recombine with its parent molecule, it recombines with a neighbouring molecule (see Fig. 3a for illustration). This set is denoted as NN trajectories. Owing to delocalization of the hole (highest-occupied molecular orbital (HOMO)) across neighbouring molecules, as visible from the results of our molecular dynamics simulations (see Supplementary Section 1 and illustration in Fig. 4e,g), such recombination on neighbouring molecules is allowed; however, typical dipole transition matrix elements are smaller than those for recombination with the parent molecule, suggesting a suppressed emission. Besides the intuitive exponential suppression, NN trajectories would also exhibit the diminished ellipticity decay with secondary peaks presented in Fig. 4, as neighbouring molecules are generally present in all spatial directions around the parent molecule. Mathematically, we can obtain such trajectories by requiring that *x*(*t*_f_) = *x*_NN_, where *x*_NN_ is a typical intermolecular separation distance (different from *l*_max_). Calculating the energy upon recollision directly provides time–frequency plots. Comparing the ab initio calculated time–frequency characteristics shown in Fig. 3b–d with those of NN trajectories (red triangles), we see a downshift of ~10 eV or more in the cut-off energy, which cannot be accounted for and generally appears in all liquids under various explored conditions. NN trajectories thus seem to mostly contribute to the onset of the second plateau and inter-plateau region, but do not have sufficiently energetic electrons to account for the full second-plateau harmonics with highest photon energies.

Another option is a set of trajectories denoted as backscattered (BS) electrons (as explored in certain solid systems^42,72^). Here we consider coherent elastic scattering of electrons after they have travelled the MFP, but whereby the BS electron can recombine with the parent molecule to emit a high-energy photon. Mathematically, these trajectories are an extension of the NN trajectories in a multi-step procedure. (i) The electron is photo-excited into the liquid-dressed continuum at *t* = *t*_i_ (at the origin with zero velocity). (ii) The quasi-free electron is semi-classically accelerated by the laser (within the SFA) up to *l*_max_, whereby it scatters coherently and elastically at *t* = *t*_s_, that is, *v*(*t*_s_) → −*v*(*t*_s_). (iii) The semi-classical dynamics continues under the laser field until a time *t*_f_, where the electron may return to the origin and recombines as a final step. The underlying dynamics can be expressed as

2 $$\begin{array}{rcl}{x}_{\rm{BS}}(t)&=&{l}_{\rm{max}}-(t-t_{s})v_{s}+\frac{q{E}_{0}}{m_{\rm{e}}{\omega }^{2}}\left[\cos (\omega t)-\cos (\omega {t}_{{\rm{s}}})\right.\\ && \left. +\omega (t-{t}_{{\rm{s}}})(\sin (\omega {t}_{{\rm{s}}}))\right],\end{array}$$

where *t*_s_ is the time when scattering occurs (at *x*(*t*_s_) = *l*_max_), which can be obtained from equation (1), and *v*_s_ is the velocity of the electron prior to backscattering at $t_{\rm{s}}, v_{\rm{s}}=\dot{x}(t_{\rm{s}})$. The recollision condition is then *x*_BS_(*t*_f_) = 0. The cross section for such intricate dynamics is expected to be very small, supporting the exponentially diminished yield of the second plateau. Moreover, this process would also support a reduced ellipticity decay in the HHG yield. However, in analysing the BS trajectories, the obtained potential HHG emission energies are again substantially too small to account for the second-plateau emission (Supplementary Section 2). Moreover, the time–frequency characteristics of BS trajectories extremely differ from the ab initio simulations (Fig. 3), suggesting that BS trajectories play almost no role in the second plateau HHG emission from the examined liquids.

We also considered the option that laser-assisted electron scattering may contribute to some of these mechanisms, thereby extending the energy cut-off closer to the measured ranges. However, such laser-assisted mechanisms inherently do not provide enough additional energy to explain the second plateau. Moreover, we remark that our ab initio simulations rely on an adiabatic approximation, which does not include electron–electron scattering effects. While this is usually regarded as a deficiency of the method, it helps here to establish that electron–electron scattering (laser assisted or not) is not responsible for the observed second HHG plateau in liquids.

Lastly, we consider trajectories of electrons recombining on the NNN molecules, that is, within the second solvation shell. Such trajectories are mathematically equivalent to the NN ones, but demanding recombination at the distance of the second disperse peak of the structure function of the liquid (for example, extending from about 4 Å to 5.5 Å in water). While the cross section for NNN recombination is weak (exponentially suppressed), it is non-zero (see illustration in Fig. 4 showing molecular dynamics simulations for hole delocalization and further discussion in Supplementary Sections 2 and 3). Energetically, NNN trajectories account for the correct energy range in the second plateau as the electrons are accelerated for a longer period of time. They also qualitatively match the ab initio time–frequency plots (Fig. 3), although the agreement is not quantitative with ~3 eV missing in the case of water (Fig. 3b) and some features not perfectly reproduced. Also note that the NNN recombination times can depend on the effective mass of the electron, which can differ in the liquid owing to screening effects. Thus, we artificially shifted the time axis for these trajectories in Fig. 3 by few hundred attoseconds to optimally match the numerical results. Importantly, NNN trajectories inherently allow for the reduced yield decay with driving ellipticity and also provide very weak cut-off scaling with respect to the driving wavelength and intensity as measured and obtained from ab initio theory, such that overall they constitute the most reasonable mechanistic explanation for the second-plateau emission. We note that we assumed here fixed-in-space holes, which is justified in liquids, given that their effective mass is high^73^.

**Extended Data Fig. 6:**
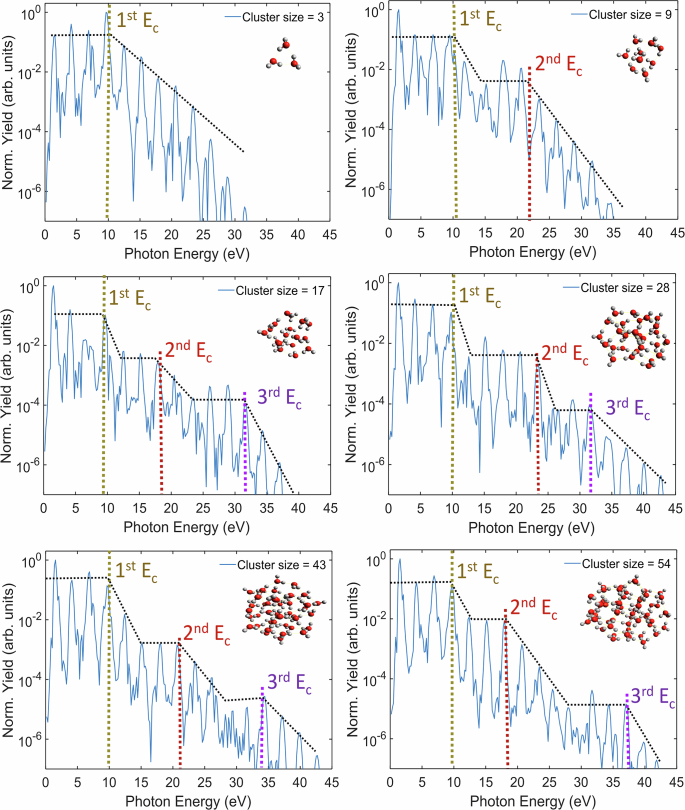
Emergence of multiple plateaus with increasing cluster size. TDDFT-calculated high-harmonic spectra of water clusters at 900 nm (5 × 10^13^Wcm^−2^). High-harmonic response of water clusters of different sizes, showing the systematic emergence of multiple plateaus as additional solvation shells form. For a 3-molecule cluster, where each molecule has only a nearest neighbor (NN) but no next-nearest neighbor (NNN), only a single plateau is observed. With increasing cluster size (9 − 54 molecules), higher-order plateaus appear progressively, with a clear second plateau emerging in the range of ~ 9 − 17 water molecules.

To further validate this picture, we developed an extended Lewenstein model^16^ (Supplementary Section 3) based on a delocalized hole resembling the HOMO obtained from molecular dynamics simulations. This model reveals that the delocalization of the hole directly leads to broader ellipticity-dependent HHG profiles and to secondary peak formation similar to that observed in the experiment and simulations in Fig. 4. As an additional test, we performed TDDFT calculations on water clusters of systematically increasing size (Extended Data Fig. 6). This approach enables a controlled inclusion of NN, NNN and higher-order solvation environments. For a three-molecule cluster, where each molecule has only an NN but no NNN, we obtain a clean single-plateau spectrum. As the cluster size increases, additional plateau structures appear, with a pronounced second plateau emerging in the range of ~9–17 water molecules. Although the exact onset requires convergence towards the bulk limit, these results demonstrate that the development of extended solvation shells—and hence the availability of off-site recombination channels—correlates directly with the appearance of secondary plateaus. These results, together with the ellipticity dependence and time–frequency analysis, identify off-site recombination as the predominant contributor to the multi-plateau dynamics, with NNN trajectories playing an important role towards the second plateau cutoff.

As a whole, these families of electronic trajectories (MFP-limited, NN, NNN and BS) paint a more complete picture of potential HHG mechanisms from liquids, as well as higher-order responses combining various scattering and recombination (on-site/off-site) processes (which might explain also the ab initio predictions of higher-order plateaus). All of these mechanisms provide cut-off scaling properties consistent with experiments and calculations (that is, near independence of laser intensity and wavelength; Fig. 3). Therefore, a distinction of dominant trajectories cannot be achieved by simple scaling measurements.

Overall, a clear physical picture for coherent laser-driven electron dynamics in liquids emerges: MFP-limited trajectories with on-site recombination dominate the first plateau, up to the first cut-off where electron–molecule scattering starts playing a role in suppressing longer and more energetic trajectories. The exponential-decay region in between plateaus and second plateau onset is contributed from those suppressed longer trajectories, and from NN trajectories that are three-step-like, but recombine off-site. An extended second plateau then emerges from coherent NN and NNN trajectories recombining on the first and second solvation shells. BS trajectories seem to be excluded under these conditions (as well as laser-assisted scattering mechanisms). Such dynamics might involve more distant recombination sites and/or higher-order scattering, possibly leading to additional plateaus visible in ab initio simulations (for example, Fig. 1d). We note that minor differences are observed between D_2_O and H_2_O (Extended Data Figs. 1 and 2), in accordance with their slight difference in structure^74^.

## Discussion and conclusion

We have performed a thorough analysis of HHG in liquids using experimental, numerical and theoretical methods. We experimentally observed the appearance of a second, exponentially suppressed, HHG plateau arising in multiple liquids and a range of laser conditions. We showed that this phenomenon is general to the liquid phase, and universal in the sense that higher-order plateaus are predicted by ab initio calculations on all studied liquids. We have used semi-classical trajectory-based models for analysing the laser-driven electron dynamics and resulting HHG, finding that the second plateau is most likely dominated by coherent emission from electrons recombining on molecules located in the first and second solvation shells. This result was supported by agreement with experimental and numerical data in terms of HHG energy and temporal characteristics, as well as additional experiments and theory in elliptically driven HHG. We identified the role of electrons recombining at the first solvation shell, and suppressed on-site recombining long trajectories, as contributing to the inter-plateau decay region, with BS electrons and laser-assisted mechanisms negligible in our examined conditions and liquids.

Let us now discuss the implications of this work. In terms of the field of HHG, we have shown that HHG in liquids can have a different mechanism compared with other phases of matter. Higher-order scattering and off-site recombination can thus potentially be probed by tracking HHG yields and cut-off energies, constituting a new spectroscopic technique in solution. This could potentially be used to determine effective solvation radii or the extent of spatial delocalization/hybridization of electronic wavefunctions between solute and solvent molecules^61^. Exploiting the built-in attosecond temporal resolution of HHG, our results may lead to the development of novel ultrafast spectroscopies for liquid-phase dynamics, including charge migration^18^, proton transfer^20^ and quantum-nuclear effects^67,75,76^, for example, exploiting isotopic substitution as means to separate ultrafast electronic from proton-transfer dynamics. Our work also suggests that attosecond pulses with higher photon energies than previously assumed can be generated, which includes isolated attosecond pulses when few-cycle driving fields are used^65^. Although we focused on liquid-phase HHG, similar phenomena are expected to arise in amorphous solids, to which the present mechanisms and results should translate, although different characteristics of the secondary plateaus can be expected. Overall, our work motivates further studies on ultrafast and nonlinear optical spectroscopies in solution and disordered phases of matter.

## Methods

### Experimental details

The experiments were performed over a wavelength range of 1,200–1,800 nm obtained by optical parametric amplification of a 1 kHz Ti:sapphire laser delivering ~30 fs pulses centred at 800 nm. The laser beam is focused with a spherical mirror onto a micrometre-thin liquid flat-jet target to generate high-order harmonics, with details provided in refs. ^4,62^. The emitted harmonics are detected in a custom-built extreme ultraviolet spectrometer composed of an aberration-free flat-field grating (SHIMADZU) and an MCP detector coupled to a phosphor screen. A CCD camera is used to image the phosphor screen. Each spectrum is typically integrated over 200 ms and averaged over 30 measurements.

The key methodological improvements in this work lie in the significant reduction of scattered light reaching the MCP detector, thereby enhancing the signal-to-noise ratio and enabling the observation of the second plateau. While the overall experimental set-up remains largely consistent with our previous work, several targeted modifications were introduced to suppress background contributions more effectively. The entrance slit was narrowed (Fig. 1a) to reduce stray light and improve spectral resolution. In addition, a new optical blocker was placed immediately after the grating stage to intercept residual scatter, primarily originating from intense first- and second-order diffraction components. To further improve sensitivity to the weaker high-order harmonics, particularly at longer driving wavelengths where the harmonic yield decreases, the MCP bias voltage was increased to −1.67 kV for 1,800 nm and −1.7 kV for 1,500 nm, compared with −1.6 kV in previous measurements. Collectively, these refinements substantially improved the detection of higher-order spectral features.

For the ellipticity-dependent measurements, the 1,800 nm light is elliptically polarized using a combination of a rotating half-wave plate (HWP) and a fixed quarter-wave plate (QWP). Our experimental geometry ensures a fixed axis of the polarization ellipse during variation from linear polarization (*ϵ* = 0) to circular polarization (*ϵ* = 1) with the rotation of the HWP axis from 0° to 22.5°, with respect to the QWP axis.

### TDDFT simulations

All TDDFT calculations were performed with the open-access code, Octopus^69^, using a real-space grid representation. For liquids, we followed the cluster approach, developed and extensively detailed in ref. ^59^, in all TDDFT simulations. In these simulations we calculated the liquid’s nonlinear response to external intense laser driving, where the driving field duration was eight cycles of the fundamental period. In almost all simulations in the main text, the dipolar response, **d**(*t*), was extracted following linearly polarized laser excitation, implementing orientation averaging following details in ref. ^59^. From **d**(*t*), we obtained HHG spectra by taking two time derivatives, and Fourier transforming, following a standard procedure, where we also filtered the dipole acceleration with a super-Gaussian window function. For simulations that involved elliptically polarized driving, a similar approach was followed, but as a result of the reduced symmetry of the light–matter Hamiltonian, many more orientations needed to be taken into account in the angular-averaging procedure (56 as opposed to 12 in the case of water). From the HHG spectra, we extracted the ellipticity-dependent HHG yield upon integrating the yield for each harmonic order. For the case of gas-phase water, we followed the same procedure, but for a single molecule response, as detailed also in refs. ^53,59^, and keeping the same level of theory as the liquid system (but with an added self-interaction correction term^77^). For Fig. 3, the Gabor transform was obtained using an exponential window function with a temporal width of a third of an optical cycle of the fundamental.

### Semi-classical trajectory simulations

For the various Newtonian equations of motion described in the main text, we numerically solved transcendental equations describing recombination conditions in each case. In all cases we assumed throughout that the electron’s effective mass is unity, and that the electron tunnel-ionizes instantaneously and with a zero initial velocity at the molecular centre (similar to the gas-phase standard approach). For liquid water, NN length was taken as the first sharp peak of the O–O distribution function at ~2.8 Å. The NNN distance was taken as the edge of the second broad peak of the distribution function at ~5.3 Å. The effective MFP (*l*_max_) in water was taken to be ~3.2 Å. The time axis was shifted by ~0.1 T in each case to compensate for potential effective-mass effects that were not explicitly included, and to obtain better agreement between the semi-classical theory and ab initio simulations. We allowed in all cases the trajectories to also surpass the original parent ionization site once, creating the two branches in the NN and NNN trajectories shown in Fig. 3 (with one branch surpassing the ion, and the other not). The kinetic energies upon recombination were obtained directly from the numerical solution of the Newtonian equations of motion, where the ab initio simulations were subtracted by the gap energy in the cluster model. In Supplementary Sections 2 and 3, we further developed a semi-analytic description of the second-plateau cut-off energies for NN trajectories, as well as a Lewenstein-like SFA numerical model^16^ for a delocalized system for the elliptically driven case.

## Online content

Any methods, additional references, Nature Portfolio reporting summaries, source data, extended data, supplementary information, acknowledgements, peer review information; details of author contributions and competing interests; and statements of data and code availability are available at 10.1038/s41566-025-01805-y.

## Supplementary information

Supplementary Information Supplementary Figs. 1–11, Sections 1–7 and Table 1.

## Data Availability

Data collected and analysed during the current study are available in the ETH Data Collection, accessible via the following link: 10.3929/ethz-c-000784683.

## Code availability

The Octopus package used for the TDDFT calculations is publicly available. The remaining computer codes are available from the corresponding authors upon reasonable request.
