# Supplementary material for: Multi-plateau high-harmonic generation in liquids driven by off-site recombination
Source: Nat Photonics. 2025 Nov 19;20(2):216–24. doi: 10.1038/s41566-025-01805-y (PMC12875872; doi:10.1038/s41566-025-01805-y)
Supplement: Supplementary file 1 — Supplementary Figs. 1–11, Sections 1–7 and Table 1. [file 41566_2025_1805_MOESM1_ESM.pdf]

nature photonics

Supplementary information https://doi.org/10.1038/s41566-025-01805-y

Multi-plateau high-harmonic generation in liquids driven by off-site recombination

In the format provided by the authors and unedited

Supporting Information for
Multi-plateau high-harmonic generation in liquids driven by off-site recombination

Angana Mondal,[1,2] Ofer Neufeld,[3,4,2,*] Tadas Balciunas,[1] Benedikt Waser,[1] Serge Müller,[1] Mariana Rossi,[4] Zhong Yin,[1,5] Angel Rubio,[4,6] Nicolas Tancogne-Dejean,[4,†] and Hans Jakob Wörner[1,‡]

[1] *Laboratorium für Physikalische Chemie, ETH Zürich, Zurich, Switzerland*
[2] *These authors contributed equally*
[3] *Technion Israel Institute of Technology, Faculty of Chemistry, Haifa 3200003, Israel*
[4] *Max Planck Institute for the Structure and Dynamics of Matter and Center for Free-Electron Laser Science, Luruper Chaussee 149, 22761 Hamburg, Germany*
[5] *International Center for Synchrotron Radiation Innovation Smart, Tohoku University, Sendai, Japan*
[6] *Center for Computational Quantum Physics (CCQ), The Flatiron Institute, 162 Fifth Avenue, New York NY 10010, USA*

S1. MOLECULAR DYNAMICS SIMULATIONS

The dynamics of pure water at normal density (0.997 g/mL) and room temperature (300 K) were run with a committee model based on high-dimensional neural network potentials (HDNNP) [1] published in Ref. [2]. We used the potentials trained on revPBE-D3 data. The water box contained 111 water molecules and was thermalized in the NVT ensemble with a stochastic velocity rescaling thermostat. The path-integral molecular dynamics were run with 32 beads and the PILE-L thermostat [3]. All dynamics were run with the i-PI code [4] interfaced with the CP2K module for HDNNP potentials [5]. From these simulations, 50 uncorrelated configurational snapshots with classical nuclei and 50 uncorrelated snapshots with quantum nuclei were randomly selected and the HOMO and LUMO orbitals were then calculated from a single-point calculation with the PBE exchange-correlation functional and *tight* species defaults in the FHI-aims code [6].

Following Ref. [7], we define the spread of the HOMO and LUMO states in terms of the first moment of their density,

$$M = \frac{\int d\mathbf{r}\rho_i(\mathbf{r}-\mathbf{r_i})|\mathbf{r}-\mathbf{r_i}|}{\int d\mathbf{r}\rho_i(\mathbf{r})}\,, \tag{1}$$

where $\mathbf{r_i}$ is the center of mass of the corresponding state.

We obtain from our different simulations:

	Quantum nuclei		Classical nuclei	
	Mean	Std. Error	Mean	Std. Error
HOMO	1.974	0.135	2.212	0.083
LUMO	9.494	0.126	9.682	0.109

TABLE I. Values for the first moments (defined in Eq.1) of the HOMO and LUMO orbitals averaged over 50 snapshots of room-temperature simulations with classical and quantum nuclei. Values are in Ångstroms.

* ofern@technion.ac.il
† nicolas.tancogne-dejean@mpsd.mpg.de
‡ hwoerner@ethz.ch

These results clearly show that the HOMO states of the various snapshots are well-localized in liquid water, while the LUMO states are fully delocalized, as clearly seen from the snapshots shown in Fig. 4 of the main text.

S2. ANALYTICAL CUTOFF LAW FOR NEAREST-NEIGHBOR RECOMBINATION

In this section, we derive a cutoff law for the nearest-neighbor recombination channel discussed in the main text (which also holds for the next-nearest-neighbor case). For this purpose, we start from the semiclassical equation of motion in one dimension, assuming a linearly polarized laser

$$x(t) = \frac{qE}{m\omega^2}[\cos(\omega t_i) - \cos(\omega t) - \sin(\omega t_i)\omega(t-t_i)], \tag{2}$$

$$v(t) = \frac{qE}{m\omega}[\sin(\omega t) - \sin(\omega t_i)]\,, \tag{3}$$

where we assumed that the trajectories start at $x(t_i) = 0$ with a vanishing velocity ($v(t_i) = 0$). Here E is the electric field strength, ω its frequency, and q and m are, respectively, the charge and mass of the electron. In the following, as well as in the next section, we assume that the parent ions are not moving on the timescale of one optical cycle. This is justified by the simulation results obtained from real-time TDDFT coupled with Ehrenfest dynamics [8] or coupled to non-adiabatic dynamics [9].

In order to evaluate the link between the energy cutoff and the distance of the nearest neighbor, we need to solve the recombination condition $x(t_r) = \pm d_{\mathrm{NN}}$, where d_{NN} is the distance between the parent ion and the recombination center. From the equation of motion, we have the condition linking the ionization time t_i to the recombination time t_r

$$\pm d_{\mathrm{NN}} = \frac{qE}{m\omega^2}[\cos(\omega t_i) - \cos(\omega t_r) - \sin(\omega t_i)\omega(t_r - t_i)]\,. \tag{4}$$

From the recombination time, we obtain the gain in kinetic energy for a trajectory initiated at t_i and returning

at t_r

$$\Delta E_K(t_i) = 2U_p\Big[\sin(\omega t_r) - \sin(\omega t_i)\Big]^2 . \quad (5)$$

However, compared to the usual case, there are some subtleties. It is clear that here not all trajectories are allowed in the sense that not all initiated trajectories will reach a NN site. In order to determine which trajectories contribute, we first need to determine for a given trajectory what is its maximum excursion distance.
The maximum excursion for the first half cycle is reached with the condition $\partial_t x(t) = v(t) = 0$. This gives us a general condition defining the time of maximal excursion t^*:

$$\frac{qE}{m\omega}[\sin(\omega t^*) - \sin(\omega t_i)] = 0, \quad (6)$$

which has an analytic solution $\omega t^* = \pi - \omega t_i + 2n\pi$. We therefore have an exact expression for the excursion distance l_{1exc} for the first half cycle of a trajectory initiated at a given ionization time:

$$l_{1exc} = |x(\pi - \omega t_i)| = \frac{qE}{m\omega^2}[2\cos(\omega t_i) - \sin(\omega t_i)(\pi - 2\omega t_i)] . \quad (7)$$

A. Direct NN recombination

For every trajectory with $l_{1exc} \geq d_{\rm NN}$, we can have direct recombination to the NN. These trajectories are denoted as direct NN trajectories, and are obtained by solving Eq. (4) and evaluating the corresponding kinetic energy upon recombination.
As shown in Fig. S1, only short duration trajectories contribute to the direct NN pathway, and their energy is lower than the gas-phase on-site recombination cutoff of $3.17U_p$, suggesting that these trajectories are not contributing to the second plateau.

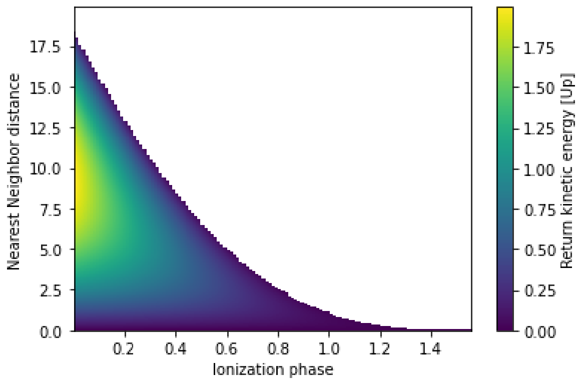

FIG. S1. Plot obtained using $\lambda = 900\,$nm and $I_0 = 7 \times 10^{13}$W.cm^{-2} for the numerical solution of all possible direct NN trajectories.

B. Indirect NN recombination

However, it is possible to have another type of NN recombination. The next possibility (in terms of number of missed scattering events) is a recombination to a NN initiated at the second half cycle. For this, we now evaluate the second excursion distance, which is for the next half-cycle and given by $\omega t^{**} = 3\pi - \omega t_i$. We find that:

$$l_{2exc} = |x(3\pi - \omega t_i)| = l_{exc} + \frac{qE}{m\omega^2}\sin(\omega t_i)2\pi . \quad (8)$$

With this in mind, we consider the next set of trajectories, the indirect NN pathway, in which we have $l_{1exc} < d_{\rm NN}$ and $l_{2exc} \geq d_{\rm NN}$. This gives the relation shown in Fig. S2.

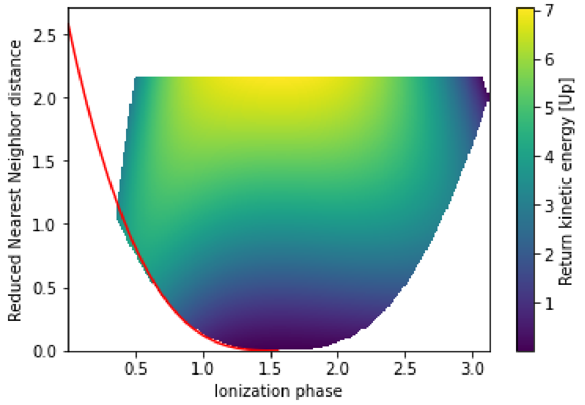

FIG. S2. Plot obtained using $\lambda = 900\,$nm and $I_0 = 7 \times 10^{13}$W.cm^{-2} and approximation for $f(\omega t_i)$ in red.

From Fig. S2 we see that the cutoff energy is potentially larger than 3.17 U_p (in agreement with the numerical simulations presented in the main text). Especially, the cutoff energy increases with the neighbor distance, as that provides the electron a longer time to classically accelerate under the effect of the laser field. Below a reduced distance $d_{NN}^{\rm red} = d_{NN}\frac{m\omega^2}{qE}$ of ≈ 0.8, the maximum kinetic energy on the edge is given by the condition

$$f(\omega t_i) = [2\cos(\omega t_i) - \sin(\omega t_i)(\pi - 2\omega t_i)] = d_{NN}^{\rm red} . \quad (9)$$

Solving this equation gives the ionization time of the most energetic trajectory $t_i^*(d_{NN}^{\rm red})$, from which we obtain the kinetic energy using Eq. 5, based on the return time found by Eq. 4.

In order to obtain an analytical approximate solution to this transcendental equation, we employ a Taylor expansion around $\pi/2$,

$$\lim_{x\to\frac{\pi}{2}} f(x) = -\frac{2}{3}(x - \frac{\pi}{2})^3 + \mathcal{O}(x^3) , \quad (10)$$

yielding an approximate expression for f for values $x < \pi/2$:

$$f(x) \approx \frac{2}{3}(\frac{\pi}{2} - x)^3 . \quad (11)$$

While this overshoots the expression in the limit of vanishing ionization time (where the limit is $2 - \pi\omega t_i + (O)((\omega t_i)^2)$), for the region of interest (i.e. $d_{NN}^{\text{red}} < 0.8$), the Taylor expansion provides a very good approximation for the recombining trajectories and kinetic energies. This result is shown in Fig. S2 for the comparison to the fully numerical solution discussed in the main text.

Lastly, we can obtain an approximate link between the ionization time for the most energetic indirect NN recombination and the reduced distance:

$$\omega t_i^* \approx \frac{\pi}{2} - \left(\frac{3}{2} d_{NN}^{\text{red}}\right)^{1/3}. \tag{12}$$

We can now numerically compute the returning cutoff energy along the red line and plot it as a function of the reduced NN distance (see Fig. S3).

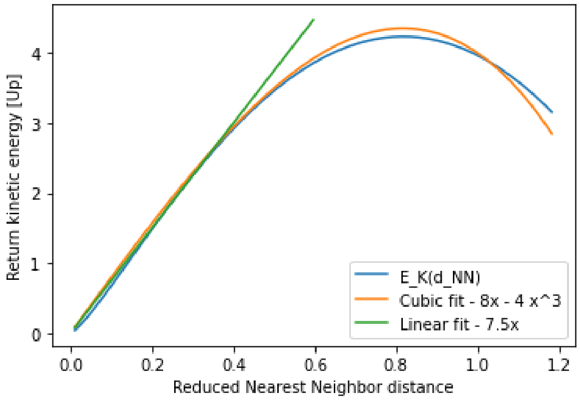

FIG. S3. Returning kinetic energy as a function of d_{NN}^{red} along the critical path, together with a linear and cubic fit

With the fit obtained, we write the approximate cutoff law

$$\Delta E_K(d_{NN}) \approx U_p(8d_{NN}^{\text{red}} - 4(d_{NN}^{\text{red}})^3) = 2qEd_{NN} - \frac{m^2\omega^4 d_{NN}^3}{qE}. \tag{13}$$

We found that the cutoff does not depend on the wavelength to first order, and that is linearly depends on the electric-field strength, in agreement with the measurements and the *ab-initio* simulations discussed in the main text.

Employing similar conditions to Fig. 3 in the main text ($\lambda = 900\,\text{nm}$, $I_0 = 7 \times 10^{13}\text{W.cm}^{-2}$, $d_{NN} = 5.5\text{Å}$), with the analytical approximation we find $d_{NN}^{\text{red}} \approx 0.6$. In this case, we find from the linear fit that the cutoff is $\sim 4.5U_p$ and from the cubic fit that it is $\sim 3.94U_p$.

S3. EXTENDED LEWENSTEIN-LIKE MODEL

We now investigate the impact of hole delocalization on liquid HHG in the context of elliptical driving fields used in the main text. For this, we need to compute the harmonic yield vs. ellipticity. We follow the work of Lewenstein et al. [10] and extend it to more complex ground-state wavefunctions that incorporate multiple possible ionization and recombination sites. Based on the molecular-dynamics results, we can conclude that (i) the HOMO state is delocalized over a few molecules, (ii) the LUMO state is fully delocalized over the entire liquid sample. This motivates employing the same approximation as typically used in the gas phase, assuming in particular that the electron evolves as a Volkov state. Subsequently, we can employ the stationary phase method to compute the integration over the initial momentum $\mathbf{p}$ of the ionized electron, and obtain the usual expression

$$\mathbf{d}(t) = \frac{1}{\sqrt{i}} \int_{-\infty}^{t} dt' \mathbf{E}(t') \mathbf{d}(\mathbf{p}(t,t') - q\mathbf{A}(t')) \frac{(2\pi)^{3/2}}{(t - t' - i\epsilon)^{3/2}} e^{-iS(\mathbf{p},t,t')} \mathbf{d}^*(\mathbf{p}(t,t') - q\mathbf{A}(t)) + c.c., \tag{14}$$

with the stationary quasi-classical action

$$S(\mathbf{p},t,t') = \int_{t_i}^{t} dt' \left(\frac{1}{2}[\mathbf{p}(t,t_i) - \mathbf{A}(t')]^2 + I_p\right). \tag{15}$$

This model can be solved numerically, provided that we readily have proper dipole matrix elements $\mathbf{d}$. These are usually taken from analytic expressions for a Gaussian or hydrogenic model of atomic-like orbitals. Here, we use a different model that allows on-site recombination, as well as off-site.

We consider a Gaussian s-orbital-like wavefunction of the form

$$\Psi(\mathbf{r}) = \left(\frac{\alpha}{\pi}\right)^{3/4} e^{-\alpha(\mathbf{r}-\mathbf{r_0})^2/2}, \tag{16}$$

with a parameter α that determines the width of the Gaussian centered around $\mathbf{r_0}$. The dipole matrix element is obtained by assuming transitions to and from plane waves, which is a typical approximation and is valid at

higher energies. After some algebra, we obtain:

$$\mathbf{d}(\mathbf{p}) = i\left(\frac{1}{\pi\alpha}\right)^{3/4} e^{-i\mathbf{p}\cdot\mathbf{r_0}} e^{-\frac{\mathbf{p}^2}{2\alpha}}\left[\mathbf{r_0} + \frac{\mathbf{p}}{\alpha}\right], \quad (17)$$

which reduces to the usual gas-phase SFA result if we take the center of the wavefunctions at the origin.

We now assume that the bound state, here the HOMO of the liquid, is composed of multiple Gaussians, with different weights and spreads, still using normalized wavefunctions. More precisely, we assume one main central Gaussian at the origin, and N surrounding identical centers with a lesser weight and a smaller spread, all located at the same distance, mimicking a liquid solvation shell. Denoting the relative weight β and the relative spread σ, this corresponds to a wavefunction of the form

$$\Psi(\mathbf{r}) = w_0\left[e^{-\alpha\mathbf{r}^2/2} + \beta\sum_{i=1}^{N} e^{-\alpha\sigma(\mathbf{r}-\mathbf{r_i})^2/2}\right], \quad (18)$$

where w_0 is an overall normalization factor.

From the previous result of the shifted Gaussian model, and using the linearity of the integral, we can express the dipole elements for this model wavefunction as

$$\mathbf{d}(\mathbf{p}) = iw_0\left(\frac{1}{\pi\alpha}\right)^{3/4}\left(e^{-\frac{\mathbf{p}^2}{2\alpha}}\frac{\mathbf{p}}{\alpha} + \frac{\beta}{\sigma^{3/4}}e^{-\frac{\mathbf{p}^2}{2\alpha\sigma}}\sum_{i=1}^{N} e^{-i\mathbf{p}\cdot\mathbf{r_i}}\left[\mathbf{r_i} + \frac{\mathbf{p}}{\alpha\sigma}\right]\right). \quad (19)$$

This expression can now be used to perform SFA simulations with the delocalized HOMO state. In order to determine the value of α, we impose that the first moment of the central Gaussian is the same as that of a hydrogenic model for which the width is given by I_p, which we take as 10eV to mimic liquid water. The values of N, β and σ are given below. Let us first compute the

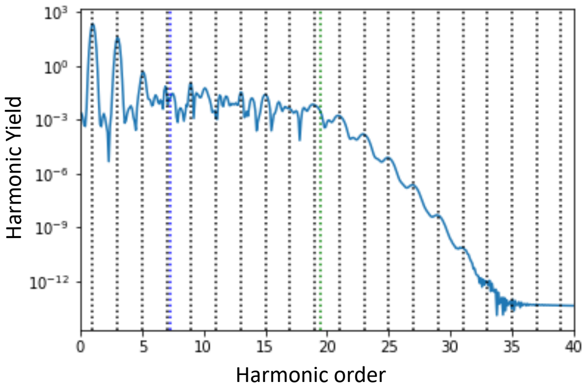

FIG. S4. HHG spectrum for the single Gaussian model.

ellipticity dependence of a single Gaussian center. The width of the Gaussian is taken such that it gives the same first moment as a hydrogenic model, with an ionization potential of 10 eV. We choose here $\lambda = 900$ nm, an intensity of $I_0 = 7 \times 10^{13}$ W.cm^{-2}, and a 6 cycle laser pulse. The HHG is computed here numerically, from the Fourier transform of the time-dependent dipole obtained from Eq. (14).
The HHG spectrum for the linearly polarized light is shown in Fig. S4.

Varying the ellipticity, we obtain the result shown in Fig. S5.

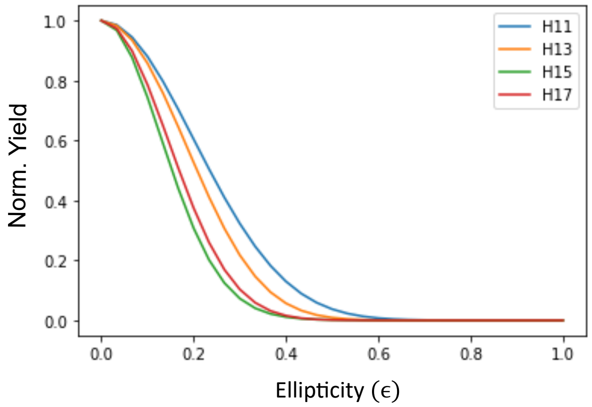

FIG. S5. Ellipticity dependence of the harmonic yield for a single-Gaussian initial state

We now perform calculations for the multi-Gaussian model. We place here 3 surrounding Gaussian at a distance of 5.5 Å around the central Gaussian, corresponding to the second solvation shell of liquid water, with $\beta = 0.01$, and $\sigma = 0.025$, which means a width of the surrounding Gaussian of 1% compared to the central one, and a FWHM divided by approximately 6.3. The positions of the Gaussian are randomly selected positions on a circle, and the time-dependent dipole is obtained from averaging over 100 random configurations of the neighbors, in order to recover the isotropic limit that is otherwise lost for an individual multi-Gaussian wavefunction of Eq. 18. Figure S6 shows that the delocalization of the hole leads to side peaks, as well as a broadening of the Gaussian profile of the ellipticity dependence. This result thus mimicks the experimentally observed data in Fig. 4 in the main text, as well as *ab-initio* simulations, and is in-line with our intuition for NN and NNN trajectories. We note that this model does not include electron scattering and mean-free path effects, as we assumed here a free propagation in vacuum, and hence cannot lead to a multiple plateau structure as observed in the experiment and extensive simulations.

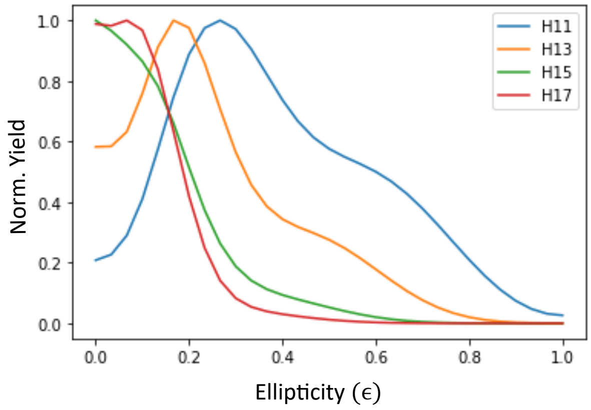

FIG. S6. Ellipticity dependence of the harmonic yield for a multi-Gaussian initial state

S4. TIME-FREQUENCY ANALYSIS FOR LIQUID AMMONIA

We further performed simulations of HHG from liquid ammonia, and performed a time-frequency analysis shown in Fig. S7, similar to what we present in the main text (Fig. 3) for liquid water and liquid methane. This result complements the data given in the main text and validates the generality of the physical mechanisms involved.

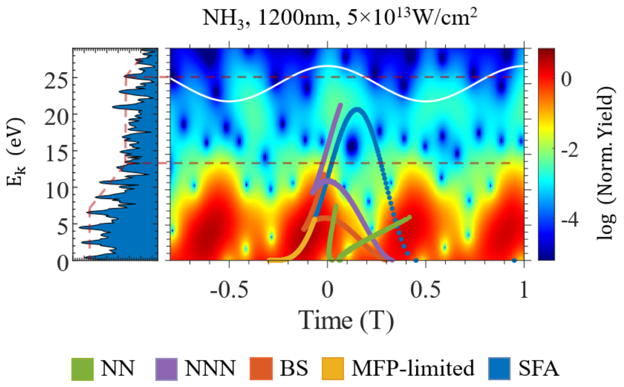

FIG. S7. Time-frequency analysis showing the showing the temporal dependence of harmonic energies for emission from liquid ammonia. Predictions from various semi-classical trajectory simulations described in the main text are superimposed, as in Fig. 3 of the main text.

S5. WAVELENGTH AND INTENSITY DEPENDENCE OF SECOND CUT-OFF

We demonstrate the experimental intensity independence of the second cut-off at multiple wavelengths (1200 nm, 1500 nm, and 1800 nm) for liquid D_2O in Fig. S8 (a-c). In all cases, the maximum intensity is capped below the plasma generation threshold. Figure S8 (d), shows the intensity independence for 900 nm H_2O from ab initio simulations. The results further confirm that the second plateau feature persists across the entire parameter range, and that the position of the second cut-off remains independent of the laser wavelength and only weakly dependent on the driving intensity, reinforcing the robustness and universality of this feature across a broad range of excitation conditions in both experiment and theory.

S6. EXPERIMENTAL AND AB INITIO ELLIPTICITY DEPENDENCE OF HARMONICS IN LIQUID WATER

The appearance of multiple Gaussian in the ellipticity dependence of the harmonic yield is observed both experimentally and theoretically in ab initio cluster simulations. For both cases (Figure S9-10), the typical gas phase single gaussian behavior of the ellipticity dependence of the harmonic yield describes the first plateau harmonics appropriately, However, we see clear appearance of multiple gaussians (beyond the single guassian feature) for second plateau harmonics. A comparison of the FWHM dependence on the harmonic energy of the liquid, derived from the single gaussian fits (Figs. S9-10), demonstrate clear deviation in behavior for the second plateau harmonics in liquids from those of their first plateau and their respective gas phases.

S7. METHODOLOGY FOR DETERMINATION OF SECOND CUT-OFF ENERGY

To accurately determine the second plateau cut-off energy (2nd E_c) in liquids, we apply two complementary methods:

1. A derivative-based approach, and

2. A piecewise linear fit intersection approach.

This dual strategy accounts for variations in plateau shapes across different liquids and ensures consistency in identifying the cut-off even when the plateau is diffuse or compressed. In gas-phase HHG, a cut-off is conventionally defined as the transition between a flat plateau and an exponential decay. However, in condensed-phase systems, especially solids and liquids, the concept of a "plateau" becomes more nuanced. Strong scattering, local disorder, and sample-dependent properties often result in plateau regions that exhibit shallow decay rather than true flatness.
For example, in solid noble gases like argon and krypton, the extent and shape of the second plateau vary with driving intensity and sample morphology [11]. In α-quartz, a narrow and flat second plateau exists (21–25 eV), whereas crystalline quartz displays a more complex

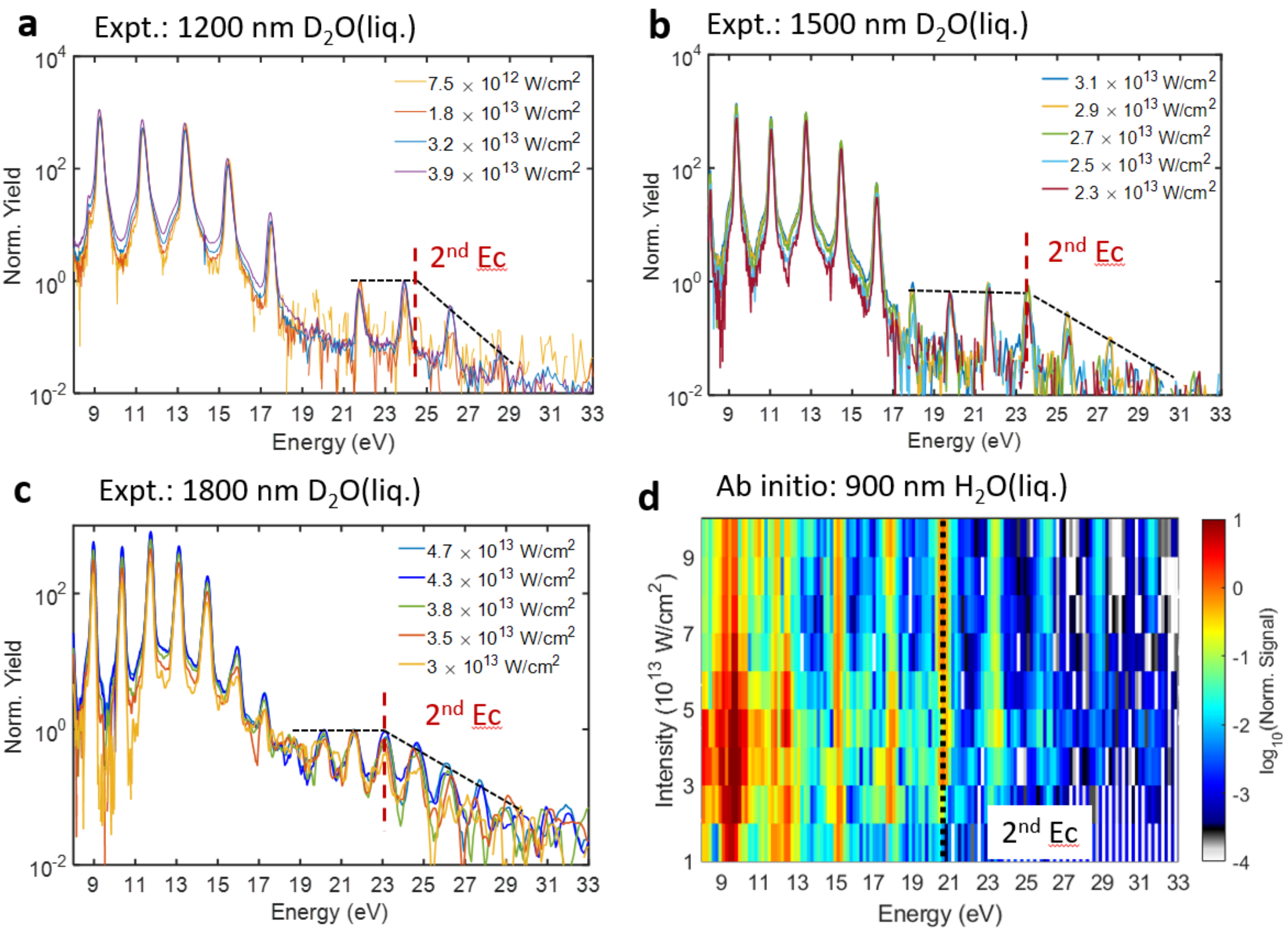

FIG. S8. Normalized HHG spectra measured in liquid D_2O at varying intensities for three different driving wavelengths: **a** 1200 nm, **b** 1500 nm, and **c** 1800 nm. Despite changes in intensity, the position of the second plateau cut-off (2nd Ec), marked by the red dashed line, remains essentially unchanged across all cases. This confirms the weak intensity dependence of the second cut-off energy. The black dashed lines are included to guide the eye through the second plateau and post-cutoff regions. **d** Ab initio simulation of HHG in liquid H_2O at 900 nm, showing the log-scaled normalized harmonic yield as a function laser intensity. The 2nd Ec is indicated by the black dashed line, beyond which the signal drops sharply across all intensities.

"double hump" structure where a precise second cut-off is ambiguous [12]. In polycrystalline and amorphous solids, this second plateau is often absent altogether.

Liquids, being structurally disordered like amorphous solids, face similar challenges. Variations in density, molecular orientation, and scattering make the plateau features highly sample-dependent. Accordingly, our methodology focuses not only on absolute yield levels but on changes in slope behavior to identify the second plateau and its termination. To illustrate the determination of the second plateau cut off energy two limiting cases of second plateau characteristics, we refer to two representative examples.

A. Liquid D_2O (Also representative of Water)

For D_2O, a clear second plateau is observed between 18–23 eV, followed by an exponential decay. In the derivative-based approach, the logarithm of the harmonic yield is interpolated with a 0.25 eV step size. The first derivative (Fig. S11 b and e) reveals a steep slope in the first plateau (<18 eV), a flattened region in the second plateau (18–22 eV), and two local minimum at 22.5 eV and ∼ 24.4 eV, respectively. To consistently identify the second cut-off energy, we define it as the deeper minimum in the derivative curve that occurs after the maximum slope value (i.e., after the shallow region). This is indicated by the red dashed lines in Figs. S11 a, b, d and e.

In the piecewise linear fit approach, the intersection of the two regions: (1) the average of the second plateau

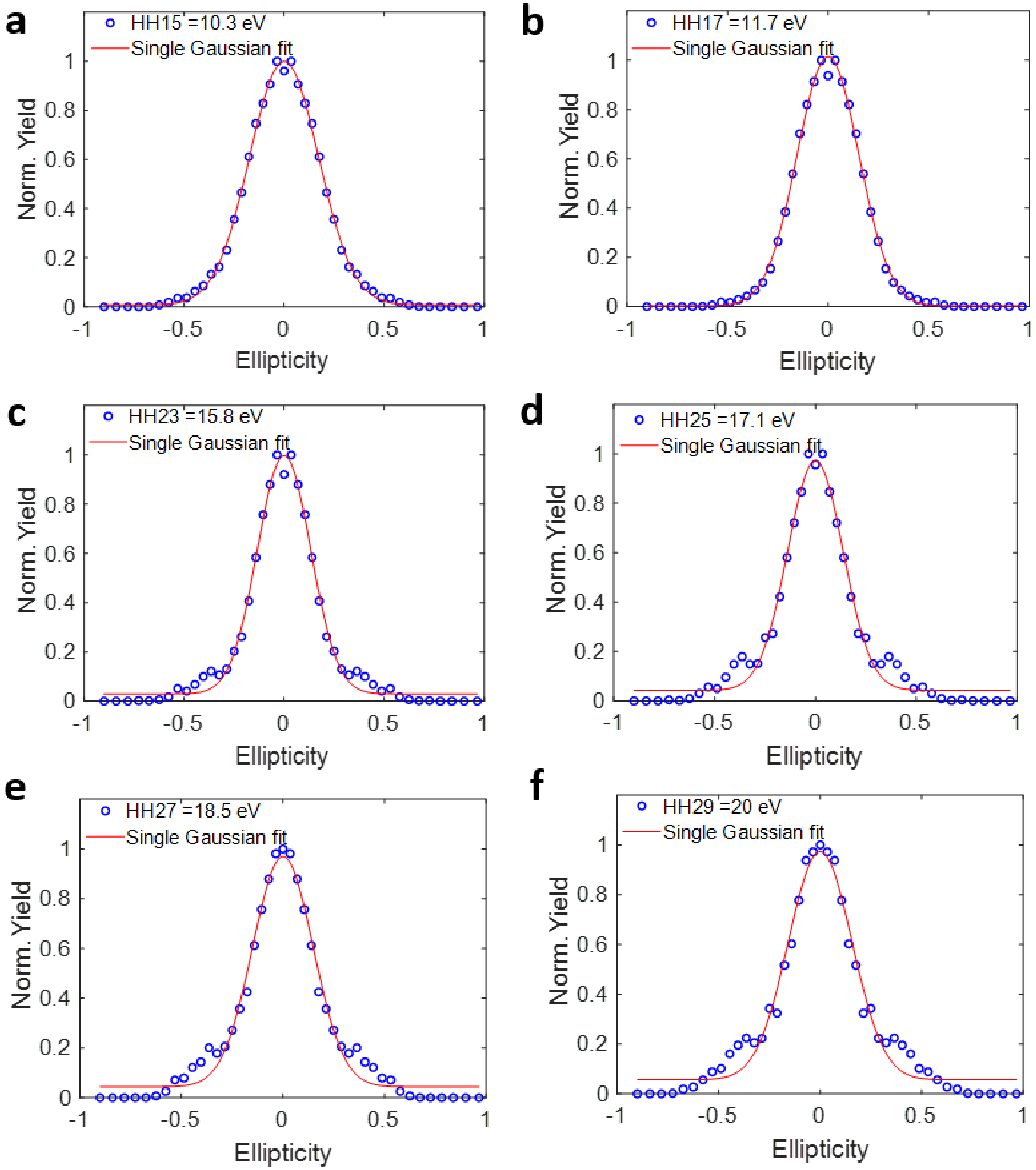

FIG. S9. Experimentally observed ellipticity dependence of harmonics for liquid ethanol using 1800 nm wavelength and their single gaussian fits.

yield (purple dashed lines in Fig. S12 c and f), and (2) the linear fit of post-plateau decay (green dashed lines in Fig. S12 c and f), defines the second cut-off (black dashed lines in Fig. S12 a, c, d, and f).

Across 1800 nm and 1500 nm driving wavelengths, both methods yield consistent cut-off energies (22.5–24.4 eV).

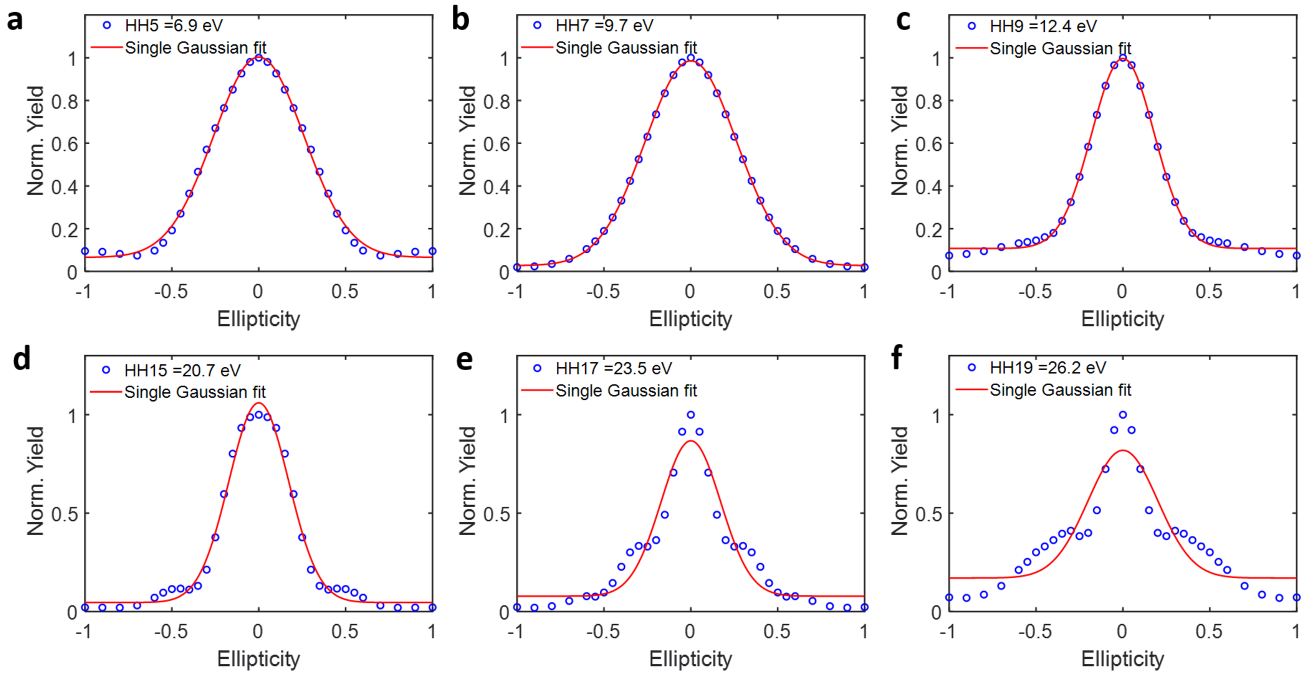

FIG. S10. Ellipticity dependence of harmonics obtained from ab initio simulations for liquid water at 900 nm and their single gaussian fits.

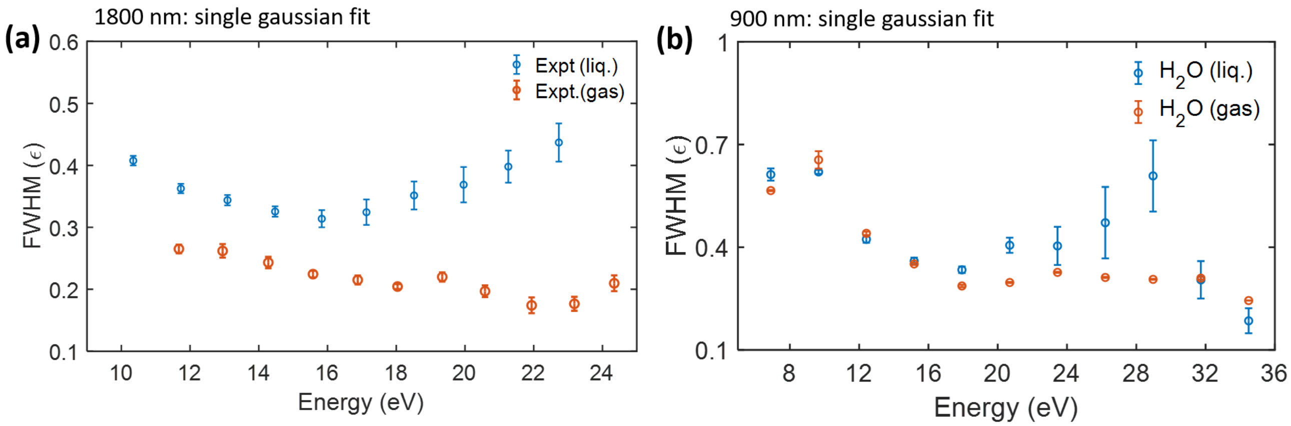

FIG. S11. Ellipticity-dependent of FWHM of harmonic yields from single-Gaussian fits. (a) Experimental results at 1800 nm comparing gas-phase (blue) and liquid-phase (orange) ethanol. (b) Ab initio simulation results at 900 nm for H2O in gas (orange) and liquid (blue) phases. Error bars in panels (a), and (b) indicate the uncertainties of the fitted parameters within a 95% confidence interval.

The final value is reported, as the average of the four cut-off energy values, at 23.5 ± one harmonic order, accounting for resolution and spectral discreteness.

B. Liquid Ethanol (Representative of Alcohols)

In ethanol, a second plateau with constant yield is not clearly observable. Instead, the harmonic spectrum shows a transition in slope—from a steep decay after the first cut-off (<15 eV) to a more gradual decay beyond 16 eV (as shown in Fig. S13 a and d). In the derivative method, the global minimum following the first maximum in the slope is taken as the second cut-off, yielding 17.6–17.9 eV across 1800 nm (red dashed line in Fig. S12 b) and 1500 nm (red dashed in Fig. S13 b). In the linear fit method, the second cut-off is defined by the intersection of two exponential decay slopes—one before and one after ∼ 16 eV. The resulting second cut-off energies are 16.7 eV for 1800 nm and 18.8 eV for 1500 nm, as shown

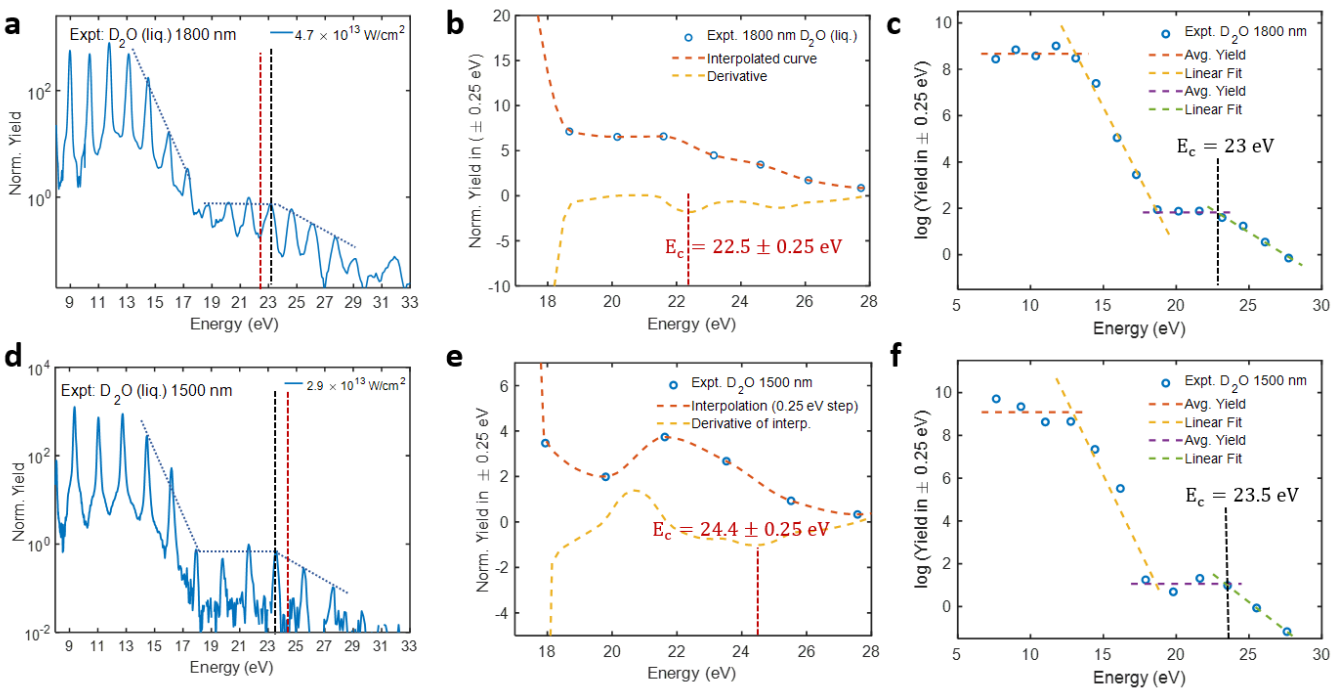

FIG. S12. Determination of the second plateau cut-off energy for liquid D2O at 1800 nm and 1500 nm. **a** Experimental HHG spectrum of liquid D2O at 1800 nm, as also shown in Extended Data Fig. 1 a. The red dashed line marks the second cut-off energy determined using the derivative-based method, while the black dashed line marks the cut-off determined via the piecewise linear intersection method. **b** Derivative-based analysis: blue circles indicate the interpolated log(harmonic yield) at 0.25 eV intervals, the red dashed line shows the interpolated curve, and the yellow dashed line represents the first derivative. The deeper minimum following the initial maximum in the derivative curve is identified as the second cut-off energy (E_c = 22.5 ± 0.25 eV). **c** Piecewise linear fit analysis: blue circles denote the same interpolated log(yield) points as in (b); the purple and green dashed lines represent linear fits to the first and second decay regions, respectively. Their intersection defines the second cut-off energy (E_c = 23 eV). (**d–f**) Same as panels **a-c** , but for the 1500 nm driving wavelength (Extended Data Fig. 2 a). The cut-off energies obtained are E_c = 24.4 ± 0.25 eV from the derivative method **e**, and E_c = 23.5 eV from the linear intersection method **f**.

by that black dashed lines in Fig. S13(c) and Fig. S13(f), respectively.
The second cut-off energy for ethanol is therefore identified, corresponding to the average of the four values, as 17.9 eV ± one harmonic order. This methodology was applied consistently across additional liquids such as H_2O and 2-propanol, and the cut-off energies were determined to be 23.4 eV ± one visible harmonic order and 17.0 eV ± one visible harmonic order, respectively. In all cases, we emphasize that assigning a cut-off energy with sub-harmonic resolution would be scientifically unjustified, as it exceeds both the experimental resolution and the discrete nature of the HHG spectrum. Thus, all reported cut-off values carry an uncertainty of ± one visible harmonic order, ensuring both rigor and physical relevance.

[1] J. Behler and M. Parrinello, Generalized neural-network representation of high-dimensional potential-energy surfaces, Phys. Rev. Lett. **98**, 146401 (2007).
[2] N. O'Neill, B. X. Shi, K. Fong, A. Michaelides, and C. Schran, To pair or not to pair? machine-learned explicitly-correlated electronic structure for nacl in water, The Journal of Physical Chemistry Letters **15**, 6081 (2024).
[3] M. Ceriotti, M. Parrinello, T. E. Markland, and D. E. Manolopoulos, Efficient stochastic thermostatting of path integral molecular dynamics, The Journal of chemical physics **133** (2010).
[4] Y. Litman, V. Kapil, Y. M. Feldman, D. Tisi, T. Begušić, K. Fidanyan, G. Fraux, J. Higer, M. Kellner, T. E. Li, *et al.*, i-pi 3.0: a flexible, efficient framework for advanced atomistic simulations, arXiv preprint arXiv:2405.15224 (2024).
[5] C. Schran, F. L. Thiemann, P. Rowe, E. A. Müller, O. Marsalek, and A. Michaelides, Machine learning potentials for complex aqueous systems made simple, Proceedings of the National Academy of Sciences **118**, e2110077118 (2021).

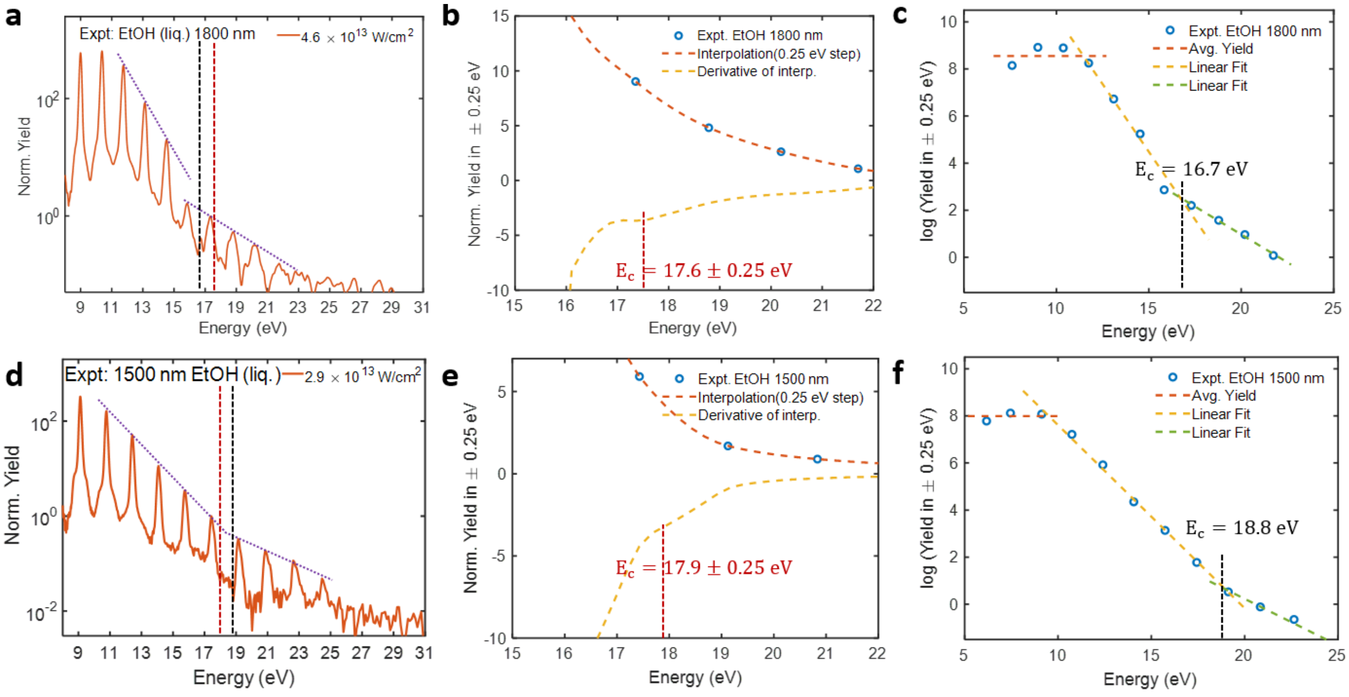

FIG. S13. Determination of the second plateau cut-off energy for liquid ethanol at 1800 nm and 1500 nm. **a** Experimental HHG spectrum of liquid ethanol at 1800 nm, as also shown in Extended Data Fig. 1 c. The red dashed line marks the second cut-off energy determined using the derivative-based method, while the black dashed line marks the cut-off determined via the piecewise linear intersection method. **b** Derivative-based analysis: blue circles indicate the interpolated log(harmonic yield) at 0.25 eV intervals, the red dashed line shows the interpolated curve, and the yellow dashed line represents the first derivative. The global minimum following the initial maximum in the derivative curve is identified as the second cut-off energy ($E_c = 17.6 \pm 0.25$ eV). (c) Piecewise linear fit analysis: blue circles denote the same interpolated log (yield) points as in **b**; the yellow and green dashed lines represent linear fits to the first and second decay regions, respectively. Their intersection defines the second cut-off energy ($E_c = 16.7$ eV). **(d–f)** Same as panels **(a–c)** , but for the 1500 nm driving wavelength (Extended Data Fig. 2c). The cut-off energies obtained are $E_c = 17.9 \pm 0.25$ eV from the derivative method **e**, and $E_c = 18.8$ eV from the linear intersection method (f).

[6] V. Blum, R. Gehrke, F. Hanke, P. Havu, V. Havu, X. Ren, K. Reuter, and M. Scheffler, Ab initio molecular simulations with numeric atom-centered orbitals, Computer Physics Communications **180**, 2175 (2009).

[7] X. Gong, S. Heck, D. Jelovina, C. Perry, K. Zinchenko, R. Lucchese, and H. J. Wörner, Attosecond spectroscopy of size-resolved water clusters, Nature **609**, 507 (2022).

[8] Z. Nourbakhsh, O. Neufeld, N. Tancogne-Dejean, and A. Rubio, An ab initio supercell approach for high-harmonic generation in liquids (2022), arXiv:2212.04177.

[9] J. Xu and S. Meng, High-harmonic generation and femtosecond-resolved ultrafast dynamics in liquid water, The Journal of Physical Chemistry Letters **16**, 5295 (2025).

[10] M. Lewenstein, P. Balcou, M. Y. Ivanov, A. L'Huillier, and P. B. Corkum, Theory of high-harmonic generation by low-frequency laser fields, Phys. Rev. A **49**, 2117 (1994).

[11] G. Ndabashimiye, S. Ghimire, M. Wu, D. A. Browne, K. J. Schafer, M. B. Gaarde, and D. A. Reis, Solid-state harmonics beyond the atomic limit, Nature **534**, 10.1038/nature17660 (2016).

[12] T. T. Luu and H. J. Wörner, Measurement of the berry curvature of solids using high-harmonic spectroscopy, Nature Communications **9**, 916 (2018).
